# Supplementary material for: Infrared characterization of formation and resonance stabilization of the Criegee intermediate methyl vinyl ketone oxide
Source: Commun Chem. 2021 Jan 22;4:8. doi: 10.1038/s42004-020-00447-1 (PMC9814936; doi:10.1038/s42004-020-00447-1)
Supplement: Supplementary file 2 — Supplementary Information [file 42004_2020_447_MOESM2_ESM.pdf]

Supplementary Information

Infrared characterization of formation and resonance stabilization of the Criegee intermediate methyl vinyl ketone oxide

Chen-An Chung[1] and Yuan-Pern Lee[1,2,3*]

[1]Department of Applied Chemistry and Institute of Molecular Science, National Chiao Tung University, Hsinchu 300093, Taiwan,

[2]Center for Emergent Functional Matter Science, National Chiao Tung University, Hsinchu 300093, Taiwan,

[3]Institute of Atomic and Molecular Sciences, Academia Sinica, Taipei 106319, Taiwan.

(Y.-P. L) - yplee@nctu.edu.tw

Table of Contents

Supplementary Note 1. Computational Results -- 1
Supplementary Note 2. IR spectra of precursor (Z)-(CH_2I)HC=C(CH_3)I (1)------------------ 1
Supplementary Note 3. IR spectra of the iodoalkenyl radical (*Z*)-$C_2H_3C(CH_3)I$ (2) ---------- 2
Supplementary Note 4. Photolysis of (*Z*)-(CH_2I)HC=C(CH_3)I (1) in O_2 at 35 Torr----------- 3
Supplementary Note 5. Spectral simulation of conformers of MVKO -------------------------- 5
Supplementary Note 6. Photolysis of (*Z*)-(CH_2I)HC=C(CH_3)I in O_2 at high pressure -------- 5
Supplementary Note 7. Temporal profiles of $C_2H_3C(CH_3)I$, $C_2H_3C(CH_3)OO$, and $C_2H_3C(CH_3)IOO$ --- 6
Supplementary Note 8. Estimated relative yields of MVKO (3) and $C_2H_3C(CH_3)IOO$ (4) -- 8
Supplementary Table 1. Cartesian coordinates of optimized geometries of precursors (*Z*)- and (*E*)- (CH_2I)HC=C(CH_3)I and iodoalkyl radicals (*Z*)- and (*E*)-$C_2H_3C(CH_3)I$ and (*Z*)- and (*E*)-(CH_2I)CHC(CH_3) predicted with the B3LYP/aug-cc-pVTZ-pp method ----------------------------- 10
Supplementary Table 2. Cartesian coordinates of optimized geometries of four conformers of the Criegee intermediates *syn-trans*-, *syn-cis*-, *anti-trans*-, and *anti-cis*-$C_2H_3C(CH_3)OO$ and dioxole predicted with the B3LYP/aug-cc-pVTZ method --- 11
Supplementary Table 3. Cartesian coordinates of optimized geometries of nine conformers of iodoperoxy radical $C_2H_3C(CH_3)IOO$ predicted with the B3LYP/aug-cc-pVTZ-pp method-------- 12
Supplementary Table 4. Cartesian coordinates of optimized geometries of six conformers of iodoperoxy radical C(CH_3)ICHCH_2OO predicted with the B3LYP/aug-cc-pVTZ-pp method ---- 14
Supplementary Table 5. Comparison of experimental vibrational wavenumbers and IR intensities of (*Z*)- (CH_2I)HC=C(CH_3)I **(1)** with those of (*Z*)- and (*E*)-(CH_2I)HC=C(CH_3)I predicted with the B3LYP/aug-cc-pVTZ-pp method-- 15
Supplementary Table 6. Vibrational wavenumbers and IR intensities of (*Z*)- and (*E*)-$C_2H_3CC(CH_3)I$ and (*Z*)- and (*E*)-(CH_2I)CHC(CH_3) predicted with the B3LYP/aug-cc-pVTZ-pp method --- 16
Supplementary Table 7. Vibrational wavenumbers and IR intensities of four conformers of Criegee intermediates $C_2H_3C(CH_3)OO$ and dioxole predicted with the B3LYP/aug-cc-pVTZ method --- 18
Supplementary Table 8. Vibrational wavenumbers and IR intensities of nine conformers of iodoperoxy radical $C_2H_3C(CH_3)IOO$ predicted with the B3LYP/aug-cc-pVTZ-pp method-------- 21
Supplementary Table 9. Vibrational wavenumbers and IR intensities of six conformers of iodoperoxy radical C(CH_3)ICHCH_2OO predicted with the B3LYP/aug-cc-pVTZ-pp method ---- 24
Supplementary Table 10. Rotational parameters and type ratios for each vibrational state of four conformers of Criegee intermediates MKVO predicted with the B3LYP/aug-cc-pVTZ method --- 26

Supplementary Table 11. Comparison of observed vibrational wavenumbers of (*Z*)-$C_2H_3C(CH_3)I$ **(2)** in region 800–1450 cm^{-1} with those calculated with the B3LYP/aug-cc-pVTZ-pp method ---------- 28
Supplementary Table 12. Comparison of observed vibrational wavenumbers of *syn-trans*-$C_2H_3C(CH_3)OO$ **(3)** in region 800–1500 cm^{-1} with those calculated with the B3LYP/aug-cc-pVTZ method ---------- 29
Supplementary Table 13. Comparison of observed vibrational wavenumbers of *syn-cis*-$C_2H_3C(CH_3)OO$ in region 800–1500 cm^{-1} with those calculated with the B3LYP/aug-cc-pVTZ method.---------- 3
0
Supplementary Table 14. Comparison of observed vibrational wavenumbers of $C_2H_3C(CH_3)IOO$ **(4)** in region 800–1500 cm^{-1} with $C_2H_3C(CH_3)IOO$-1 and $C_2H_3C(CH_3)IOO$-2 calculated with the B3LYP/aug-cc-pVTZ-pp method---------- 31
Supplementary Table 15. Summary on estimates of concentrations of species in varied experiments---------- 32
Supplementary Figure 1. Geometries of conformers of methyl vinyl ketone oxide (MVKO) and dioxole predicted with the B3LYP/aug-cc-pVTZ method---------- 33
Supplementary Figure 2. Geometries of precursors (*Z*)-/(*E*)-(CH_2I)HC=C(CH_3)I) and (*Z*)-/(*E*)-iodoalkyl radicals $C_2H_3C(CH_3)I$ and (CH_2I)CHC(CH_3) predicted with the B3LYP/aug-cc-pVTZ-pp method ---------- 34
Supplementary Figure 3. Geometries of nine conformers of iodoperoxy radicals $C_2H_3C(CH_3)IOO$ predicted with the B3LYP/aug-cc-pVTZ-pp method. ---------- 35
Supplementary Figure 4. Geometries of six conformers of iodoperoxy radicals $C(CH_3)ICHCH_2OO$ predicted with the B3LYP/aug-cc-pVTZ-pp method. ---------- 36
Supplementary Figure 5. Comparison of IR spectra of (*Z*)-$CH_2IHC=C(CH_3)I$ **(1)** in the gaseous phase and in solid *p*-H_2 with quantum-chemical calculations ---------- 37
Supplementary Figure 6. Temporal evolution of observed spectra and processed spectra in region 1450–850 cm^{-1} at resolution 1.0 cm–1 upon photolysis at 248 nm of a flowing mixture of (*Z*)-1,3-diiodo-but-2-ene/N_2 (0.03/75 Torr) and comparison with predicted stick spectra.---------- 38
Supplementary Figure 7. Comparison of IR spectra of (*Z*)-$C_2H_3C(CH_3)I$ **(2)** in the gaseous phase with quantum-chemical calculations---------- 39
Supplementary Figure 8. Temporal evolution of observed and processed spectra in region 1450–850 cm^{-1} at resolution 0.5 cm^{-1} upon photolysis at 248 nm of a flowing mixture of (*Z*)-1,3-diiodo-but-2-ene/O_2 (0.04/35 Torr).---------- 40
Supplementary Figure 9. Resonance structures and frontier orbitals of *syn*-trans-MVKO---------- 41
Supplementary Figure 10. Temporal evolution of observed and processed spectra in region 1450–850 cm^{-1} at resolution 1.0 cm^{-1} upon photolysis at 248 nm of a flowing mixture of (*Z*)-1,3-diiodo-but-2-ene/O_2 (0.042/347 Torr) ---------- 42

Supplementary Figure 11. Temporal evolution of observed and processed spectra in region 1450–850 cm^{-1} at resolution 1.0 cm^{-1} upon photolysis at 248 nm of a flowing mixture of (*Z*)-1,3-diiodo-but-2-ene/O_2 (0.035/236 Torr) -- 43
Supplementary Figure 12. Comparison of bands in group C with stick IR spectra of nine conformers of iodoperoxy radical $C_2H_3C(CH_3)IOO$ -- 44
Supplementary Figure 13. Comparison of bands in group C with stick IR spectra of six conformers of iodoperoxy radical $C(CH_3)ICHCH_2OO$ -- 45
Supplementary Figure 14. Temporal profiles of species upon photolysis at 248 nm of a mixture of (*Z*)-$(CH_2I)HC{=}C(CH_3)I$ (0.04 Torr) and O_2 (35 Torr) and a mixture of (*Z*)-$(CH_2I)HC{=}C(CH_3)I$ (0.04 Torr) and O_2 (347 Torr). -- 46
Supplementary References -- 47

Supplementary Note 1. Computational Results

The geometries and relative energies of four conformers of Criegee intermediates *syn-trans*-, *syn-cis*-, *anti-trans*-, and *anti-cis*-$C_2H_3C(CH_3)OO$ and dioxole are presented in Supplementary Figure 1. Those of precursors (*Z*)- and (*E*)-$(CH_2I)HC{=}C(CH_3)I$ and iodoalkyl radicals (*Z*)- and (*E*)-conformers of $C_2H_3C(CH_3)I$ and $(CH_2I)CHC(CH_3)$ are presented in Supplementary Figure 2. The geometries and relative energies of nine possible conformers of iodoperoxy radical $C_2H_3C(CH_3)IOO$ and six possible conformers of $C(CH_3)ICHCH_2OO$ are presented in Supplementary Figures 3 and 4, respectively. Cartesian coordinates of all conformers of $(CH_2I)HC{=}C(CH_3)I$, $C_2H_3C(CH_3)I$, $(CH_2I)CHC(CH_3)$, $C_2H_3C(CH_3)OO$, dioxole, $C_2H_3C(CH_3)IOO$, and $C(CH_3)ICHCH_2OO$ are listed in Supplementary Tables 1–4.

Computed scaled harmonic vibrational wavenumbers and IR intensities of precursors (*Z*)- and (*E*)-$(CH_2I)HC{=}C(CH_3)I$, iodoalkyl radicals (*Z*)- and (*E*)-conformers of $C_2H_3C(CH_3)I$ and $(CH_2I)CHC(CH_3)$, Criegee intermediates *syn-trans*-, *syn-cis*-, *anti-trans*-, and *anti-cis*-$C_2H_3C(CH_3)OO$ and dioxole, nine conformers of iodoperoxy radicals $C_2H_3C(CH_3)IOO$, and six conformers of iodoperoxy radicals $C(CH_3)ICHCH_2OO$ are listed in Supplementary Tables 5–9, respectively. The anharmonic vibrational wavenumbers of Criegee intermediates *syn-trans*-, *syn-cis*-, *anti-trans*-, and *anti-cis*-$C_2H_3C(CH_3)OO$ and dioxole are also listed in Supplementary Table 7. The rotational parameters and type ratios for each vibrational state of the four conformers of Criegee intermediates MKVO are listed in Supplementary Table 10.

Supplementary Note 2. IR spectra of precursor (*Z*)-$(CH_2I)HC{=}C(CH_3)I$ (1)

The IR spectrum of gaseous precursor (*Z*)-$(CH_2I)HC{=}C(CH_3)I$ **(1)** in region 1450–850 cm^{-1} is presented in Supplementary Figure 5(a). This spectrum is compared with the spectrum of **(1)** in a solid *p*-H_2 matrix[1] and stick spectra of **(1)** predicted with the B2PLYP-D3 and B3LYP methods in

Supplementary Figure 5. Four intense bands near 1434, 1294, 1152, and 1063 cm^{-1} and two weaker ones near 1384 and 1169 cm^{-1} (the latter appears as a shoulder of the band near 1152 cm^{-1}) were observed, in agreement with those observed for the same conformer in a *p*-H_2 matrix at 3.2 K.[1] According to the plot of experimental wavenumbers versus harmonic vibrational wavenumbers of **(1)** predicted with the B3LYP/aug-cc-pVTZ-pp method, we derived a linear scaling equation y = (0.9708 ± 0.0159) x + (9.3 ± 20.7), in which y and x are experimental and harmonic vibrational wavenumbers, respectively. All observed wavenumbers and intensities are compared with those observed in solid *p*-H_2 and scaled harmonic vibrational wavenumbers and IR intensities in Supplementary Tables 5. We employed this equation to scale the predicted harmonic vibrational wavenumbers of other species considered in this work. On comparison of the spectrum of precursor reported by Barber et al. (in Supporting Information),[2] the samples that these authors used were clearly a mixture of both (*Z*)- and (*E*)-conformers, with the former dominant, as they stated. In this work, a nearly pure (*Z*)-conformer **(1)** was used.

Supplementary Note 3. IR spectra of the iodoalkenyl radical (*Z*)-$C_2H_3C(CH_3)I$ (2)

When the diiodoalkene precursor **(1)** in N_2 was irradiated with light at 248 nm, the intensity of its lines decreased significantly, as shown in Supplementary Figure 6(b) as a difference spectrum obtained from the *ac*-channel recorded 0–3 μs after irradiation; intense negative bands indicate the destruction of the precursor, whereas the formation of products is indicated by some extremely weak positive features. The expanded spectra of products recorded 0–3 and 10–15 μs after irradiation are shown in Supplementary Figures S6(c) and S6(d), respectively, with the negative bands truncated. The features corresponding to the primary photolysis product decreased with time, but a broad feature near 915 cm^{-1} and two sharp lines at 919 and 892 cm^{-1} increased continuously. We termed these six features near 1406, 1261, 1109, 1019, 925, and 873 cm^{-1} that are associated with the primary photolysis product as group A and marked them A_1–A_6 in Supplementary Figure 6(c); the

bands with positions near 925 and 873 cm^{-1} are less certain because of their small intensities and severe interference from the broad feature near 915 cm^{-1}. The intensities of these features in group A decreased significantly in the spectrum recorded 10–15 μs after irradiation, indicating the transient nature of the carrier. We added the bands of the precursor, Supplementary Figure 6(a), back to the spectrum in Supplementary Figure 6(c) to minimize its interference and present it in Supplementary Figure 6(e); the regions with which the intense absorption of the precursor might interfere are shaded with grey. Some regions of the parent absorption could not be compensated completely because some precursors might become internally excited upon irradiation, so that their absorption spectrum is differed from that before irradiation.

The assignments of these new features in group A to the iodoalkenyl radical (*Z*)-$C_2H_3C(CH_3)I$ **(2)** is discussed in the main text. Comparison of IR spectra of features in group with the IR stick spectra of two possible photolysis products, (*Z*)-$C_2H_3C(CH_3)I$ **(2)** and (*Z*)-$(CH_2I)CHC(CH_3)$, according to the scaled harmonic vibrational wavenumbers predicted with the B3LYP method are shown in Figure 2. The observed new features agree satisfactorily with lines predicted near 1418, 1261, 1108, 1018, 930, and 887 cm^{-1} for **(2)**, as compared in Supplementary Table 11. Comparison of IR spectra of lines in group A with the IR stick spectra of (*Z*)-$C_2H_3C(CH_3)I$ **(2)** and (*E*)-$C_2H_3C(CH_3)I$ is presented in Supplementary Figure 7; the agreement of experiments with the latter is poor, indicating that the conversion from (*Z*)- to (*E*)-conformation did not occur.

Supplementary Note 4. Photolysis of (*Z*)-$(CH_2I)HC{=}C(CH_3)I$ (1) in O_2 at 35 Torr

The top trace in Supplementary Figure 8(a) shows the absorption spectrum, on a reduced scale, of a flowing mixture of **(1)** (0.04 Torr) and O_2 (35 Torr) before photolysis; Supplementary Figures 8(a)–(c) show expanded difference spectra recorded 0–5, 5–10, and 30–35 μs, respectively, after photolysis of the mixture at 248 nm; the negative bands corresponding to the destruction of the

precursor are truncated. The spectrum of the iodoalkenyl radical (*Z*)-$C_2H_3C(CH_3)I$ **(2)** shown in Supplementary Figure 6(e) is reproduced in Supplementary Figure 8(d) for comparison. Small bands of **(2)** were observed only 0–5 μs after irradiation before it reacted completely with O_2. Some new features appeared and their intensities reached maxima near 5–10 μs, whereas some bands appeared at a later period and became more prominent in the spectrum recorded 30–35 μs after irradiation; the latter features correspond to absorption of the end product methyl vinyl ketone (MVK, $C_2H_3C(O)CH_3$), of which spectrum is presented in Supplementary Figure 8(e) for comparison. We stripped absorption bands of the iodoalkenyl radical **(2)** and MVK and added back the bands of the precursor **(1)** in spectra shown in Supplementary Figures 8(a)–(c); the resultant spectra are presented in Supplementary Figures 8(f)–(h). Seven bands near 1416, 1383, 1346, 1060, 987, 948, and 916/908 cm^{-1} that have correlated variations in intensity and reached their maxima 5–10 μs after irradiation are termed group B and marked B_1–B_7 in Supplementary Figure 8(g); feature B_7 contains two sharp bands at 916 and 908 cm^{-1}. The regions with which intense absorption of the precursor might interfere are shaded with grey; however, perhaps only the region 1140–1180 cm^{-1} containing the most intense absorption band of the precursor is unreliable. Although feature B_4 near 1060 cm^{-1} overlaps partially with the absorption band of the precursor near 1063 cm^{-1}, its absorption contour is expected to be reliable because of the excellent reproducibility of our FTIR spectra. The weak band near 891 cm^{-1} does not belong to group B because of its unsatisfactory correlation in intensity variations in varied reaction periods. These features in group B are assigned to the Criegee intermediate *syn*-*trans*-MVKO **(3)**, as discussed in the main text. Comparison of observed vibrational wavenumbers of *syn*-*trans*-$C_2H_3C(CH_3)OO$ (**3**) in region 800–1500 cm^{-1} with those calculated with the B3LYP/aug-cc-pVTZ method is shown in Supplementary Table 12. The major resonance structures of *syn*-*trans*-MVKO **(3)** is shown in Supplementary Figure 9.

Some weak features might be tentatively assigned to *syn*-*cis*-$C_2H_3C(CH_3)OO$, as discussed in

the main text. Observed vibrational wavenumbers of *syn-ciss*-$C_2H_3C(CH_3)OO$ in region 800–1500 cm^{-1} are compared with those predicted with the B3LYP/aug-cc-pVTZ method in Supplementary Table 13.

Supplementary Note 5. Spectral simulation of conformers of MVKO

With program PGopher,[3] we simulated the rotational contours of some vibrational modes of Criegee intermediates *syn-trans*-, *syn-cis*-, *anti-trans*-, and *anti-cis*-MVKO using rotational parameters of the lower state (A'', B'', and C'') and upper states (A', B', and C'), and ratios of *a*-type/*b*-type/*c*-type predicted with the B3LYP/aug-cc-pVTZ method (Supplementary Table 10). The parameters employed in the simulations are J_{max} = 200, T = 298 K, Gaussian width (FWHM) = 0.64 (corresponding to the instrument resolution of 0.5 cm^{-1}). The weighting factors of bands of types *a*, *b*, and *c* in each resultant vibrational absorption band were determined by the squares of the projections of the dipole derivatives for each vibrational mode onto rotational axes *a*, *b* and *c*.

Supplementary Note 6. Photolysis of (*Z*)-$(CH_2I)HC{=}C(CH_3)I$ in O_2 at high pressure

The top trace in Supplementary Figure 10(a) shows the absorption spectrum, on a reduced scale, of (*Z*)-1,3-diiodobut-2-ene/O_2 (0.042/347 Torr) in a flowing mixture before photolysis. Supplementary Figures 10(a)–(c) present the difference absorption spectra of this flowing mixture 0–25, 25–50, and 50–100 μs after photolysis; the spectra were recorded with the internal 24-bit ADC with temporal resolution 12.5 μs. Supplementary Figures 10(d) and 10(e) depict reference spectra of *syn-trans*-MVKO **(3)** and MVK, respectively. Supplementary Figures 10(f)–(h) show the spectra processed from Supplementary Figures 10(a)–10(c), with absorption of MVKO **(3)** and MVK stripped and that of the precursor **(1)** added back. Similar plots for experiments of (*Z*)-1,3-diiodobut-2-ene/O_2 (0.035/236 Torr) 0–10, 10–20, and 20–30 μs after photolysis, recorded with an external ADC, and those processed with bands of MVKO **(3)** and MVK removed and those of the precursor

(1) added back are presented in Supplementary Figure 11. Six bands near 1375, 1213, 1108, 1063, 986, and 885 cm^{-1} that showed correlated intensity variations and decayed slowly after irradiation are termed group C and marked C_1–C_6 in Supplementary Figures 10(f) and 11(f). The band near 934 cm^{-1} appears not to be associated with group C because of its unsatisfactory correlation in intensity variations at varied experimental period; it might be an artifact from stripping the most-intense band of *syn-trans*-MVKO **(3)**. The observed spectra of bands of group C are compared with the stick IR spectra of nine possible conformers of $C_2H_3C(CH_3)IOO$ **(4)** in Supplementary Figure 12 and with the stick spectra of six possible conformers of $C(CH_3)ICHCH_2OO$ in Supplementary Figure 13. These features in group C are assigned to the iodoperoxy adduct $C_2H_3C(CH_3)IOO$ **(4)**, as discussed in the main text. Comparison of observed vibrational wavenumbers of $C_2H_3C(CH_3)IOO$ **(4)** in region 800–1500 cm^{-1} with those calculated with the B3LYP/aug-cc-pVTZ method for the two least-energy conformers is shown in Supplementary Table 14.

Supplementary Note 7. Temporal profiles of $C_2H_3C(CH_3)I$ (2), $C_2H_3C(CH_3)OO$ (3), and $C_2H_3C(CH_3)IOO$ (4)

Supplementary Figure 14(a) presents temporal profiles of the iodoalkenyl radical **(2)** (black triangles) and the MVKO **(3)** (blue circles) upon UV irradiation of **(1)** in O_2 at 35 Torr. Parts of band A_2 (1260–1270 cm^{-1}) and B_6 (920–960 cm^{-1}) in the raw spectra were integrated to yield the temporal evolution of **(2)** and **(3)**, respectively. The integrated intensity of the iodoalkenyl radical **(2)** showed an immediate rise upon UV irradiation of the mixture, followed by a rapid decay before it reached a non-zero baseline. The non-zero baseline is due to some contribution of the final product MVK shown in Supplementary Figure S8(e), which was unavoidable. The integrated intensity of MVKO **(3)** showed an initial rise from zero, followed by a slow decay; its rate of rise correlates well with the rate of decay of **(2)**, supporting that **(3)** was produced from the reaction of **(2)** with O_2 and that our

spectral assignments are reasonable.

Supplementary Figure 14(b) presents the temporal profiles of iodoperoxy adduct **(4)** (black triangles and red inverted triangles) and MVKO **(3)** (blue circles) in an experiment of **(1)** in O_2 at 347 Torr. Parts of bands C_3 (1100–1120 cm^{-1}), C_6 (862–907 cm^{-1}), and B_6 (920–960 cm^{-1}) were integrated to yield the temporal evolution of **(4)** and **(3)**, respectively. All profiles were normalized for ease of comparison; species **(3)** and **(4)** showed a similarly sharp rise due to the rapid formation reaction of **(2)** with O_2 at high pressure, followed by different slow decays. The similar rate of rise is consistent with the expectation from a parallel reaction, supporting that MVKO **(3)** and the iodoperoxy adduct **(4)** were produced from the same reaction, that of iodoalkenyl radical **(2)** with O_2. In contrast, the temporal profile of MVK (pink diamonds, integrated over 1250–1270 cm^{-1}) had a slow rise, indicating the nature of secondary formation. The fraction of loss of MVKO **(3)** appeared to be smaller than that of **(4)**. This might be because band B_6 overlaps with an intense band of MVK in this region, and MVK is a major product when O_2 pressure is high. The profile of **(3)** after 0.2 ms is thus unreliable and might reflect mostly the behavior of MVK. Further experiments with higher spectral resolution are needed to clarify this problem.

The spectra obtained at 35 Torr showed predominant production of **(3)** with little contribution of **(4)** (Supplementary Figure 8). In contrast, at 236 and 347 Torr, the yield of **(3)** decreased and that of **(4)** increased significantly (Supplementary Figures 10 and 11). The analysis is listed in Supplementary Table 15 and discussed in the following section. This observation is also consistent with the expectation that the iodoperoxy adduct **(4)** is stabilized at higher pressure instead of decomposition to MVKO + I, similarly to what was observed in experiments of $CH_2I + O_2$.[4] Lin et al. recently proposed that, based on their observation of the temporal profiles of UV absorption of MVKO (**3**) above 50 Torr, the iodoperoxy adduct was produced from the source reaction but decomposed to MVKO + I at a time scale of ms,[5] in support of our observation of **(4)**. We did

observe the decay of iodoperoxy adduct, but we are uncertain about the formation of MVKO **(3)** from **(4)** because the interference from the intense band of MVK, as stated previously.

Supplementary Note 8. Estimated relative yields of MVKO (3) and $C_2H_3C(CH_3)IOO$ (4)

The relative yields of MVKO **(3)** and (*Z*)-$C_2H_3C(CH_3)IOO$ **(4)** at varied pressures were estimated on integrating the destruction of a band (1130–1190 cm^{-1}) of precursor **(1)**, the formation of band B_6 (920–960 cm^{-1}) of MVKO **(3)** and bands C_3 (1100–1120 cm^{-1}) and C_6 (862–907 cm^{-1}) of $C_2H_3C(CH_3)IOO$ **(4)**; these integrated absorbance were divided with their respective IR intensities predicted with the B3LYP method. The concentration *c* (in molecule cm^{-3}) of each species except **(1)** in the photolysis region was calculated according to equation (1),

$$c = 2.65 \times \frac{2.30 \int \Delta A \ d\tilde{v}}{l \times S} \quad (1)$$

in which 2.65 is the volume ratio, V_{IR}/V_{UV}, for the IR probe and UV photolysis,[6] $\int \Delta A \ dv$ is the intensity in cm^{-1} for the integrated region in the difference spectrum (base 10), 2.30 is ln 10, *l* is 360 cm for IR path length, and *S* is the band intensity in cm $molecule^{-1}$ calculated with the B3LYP method. The partial pressure of **(1)** was derived on comparing the observed integrated absorbance of IR bands in regions 1130−1190 cm^{-1} and 1025−1085 cm^{-1} with the calibration curve obtained at varied pressures. The experimental results are summarized in Supplementary Table S15. We emphasize that the error of the calculated IR intensity might be as large as factor 2 and is not included in the listed errors. Furthermore, as some bands might suffer interference from absorption of other species, the estimated concentrations might have large errors. Nevertheless, a comparison of the relative values of the same species is expected to be reliable.

Because band C_6 of $C_2H_3C(CH_3)IOO$ **(4)** in region 862–907 cm^{-1} overlaps with the weak band

B_7 of MVKO **(3)**, we performed spectrum subtraction to remove the contribution of **(3)** and estimated the error associated with this interference to be less than 11 %. Even including this possible errors, the observed intensity of band C_6 relative to those of other bands in group C appeared, however, to be much greater than theoretically predicted; the absolute concentration of **(4)** according to band C_6 might be overestimated. As shown in rows 10 and 11 of Supplementary Table S15, the estimated concentration of **(4)** according to bands C_3 and C_6 varied by factor 1.5–2.3.

Nevertheless, the percentage variation of concentrations estimated from each band as the total pressure increased, after taking into account the difference in photolysis yield of the precursor **(1)** in each experiment, is expected to be reliable. The derived relative concentrations of **(3)** and **(4)** are compared at varied pressure. From rows 12–14 in Supplementary Table 15, it is clear that the relative yield of MVKO **(3)** decreased to ~63 %, whereas that of the iodoperoxy adduct **(4)** increased significantly to a factor 1.8–2.9, as the pressure increased from 35 Torr to 347 Torr.

Supplementary Table 1 Cartesian coordinates of optimized geometries of precursors (*Z*)- and (*E*)- $(CH_2I)HC{=}C(CH_3)I$ and iodoalkyl radicals (*Z*)- and (*E*)-$C_2H_3C(CH_3)I$ and (*Z*)- and (*E*)-$(CH_2I)CHC(CH_3)$ predicted with the B3LYP/aug-cc-pVTZ-pp method.

	x	*y*	*z*	*x*	*y*	*z*
	**(*Z*)-$(CH_2I)HC{=}C(CH_3)I$**			**(*E*)-$(CH_2I)HC{=}C(CH_3)I$**		
C_1	-0.86339	0.34407	1.17814	-1.32719	-0.66300	1.12428
C_2	-0.00616	1.38122	0.58527	0.00355	-0.08842	0.84376
C_3	1.21426	1.25485	0.06672	0.97664	-0.70146	0.16870
C_4	2.01770	2.37724	-0.50570	0.95084	-2.04267	-0.48671
H_1	-0.36188	-0.60061	1.34490	-1.68249	-0.41647	2.11797
H_2	-1.37213	0.68000	2.07420	-1.41672	-1.72564	0.94465
H_3	-0.42431	2.38418	0.56639	0.17318	0.90676	1.23172
H_4	2.96739	2.49162	0.01892	1.14757	-1.94874	-1.55573
H_5	2.24994	2.19618	-1.55652	1.72281	-2.69406	-0.07434
H_6	1.46150	3.31266	-0.42875	-0.01543	-2.53002	-0.36326
I_1	2.20124	-0.63627	-0.02399	2.83370	0.32656	-0.05947
I_2	-2.55398	-0.16766	-0.16404	-2.90072	0.22781	-0.17075
	**(*Z*)-$(C_2H_3)C(CH_3)I$**			**(*E*)-$(C_2H_3)C(CH_3)I$**		
C_1	-2.12400	-1.67023	0.00000	1.60646	2.89572	0.00000
C_2	-2.12260	-0.29766	0.00000	1.28358	1.55870	0.00000
C_3	-1.05063	0.58203	0.00000	0.00000	1.02600	0.00000
C_4	-1.18368	2.06637	0.00000	-1.28306	1.78190	0.00000
H_1	-1.20694	-2.24109	0.00000	2.64163	3.20242	0.00000
H_2	-3.05594	-2.21494	0.00000	0.86246	3.67838	0.00000
H_3	-3.09169	0.19715	0.00000	2.10591	0.85388	0.00000
H_4	-0.70936	2.51327	0.87730	-1.88661	1.53908	-0.87767
H_5	-0.70883	2.51342	-0.87730	-1.88661	1.53908	0.87767
H_6	-2.23818	2.34761	0.00000	-1.10240	2.85622	0.00000
I_1	0.94144	-0.13582	0.00000	-0.19578	-1.08006	0.00000
	**(*Z*)-$(CH_2I)CHC(CH_3)$**			**(*E*)-$(CH_2I)CHC(CH_3)$**		
C_1	-0.49186	1.12935	0.17418	0.64727	-0.74350	-0.72959
C_2	-1.68724	0.49456	-0.42624	1.65206	-0.96739	0.34563
C_3	-2.72293	0.06829	0.25386	2.76830	-0.29032	0.46848
C_4	-4.03259	-0.55200	0.06234	3.52726	0.81821	-0.10869
H_1	-0.59465	1.30843	1.23704	0.30367	-1.66665	-1.18315
H_2	-0.17982	2.02905	-0.34404	0.97040	-0.04326	-1.48991
H_3	-1.68495	0.39125	-1.51500	1.42376	-1.76235	1.05060
H_4	-4.09373	-1.51284	0.57787	3.68479	1.60691	0.63031
H_5	-4.83350	0.08020	0.45126	4.51093	0.48966	-0.45053
H_6	-4.22958	-0.72806	-1.00389	3.00671	1.26757	-0.96493
I_1	1.30611	-0.15867	0.00400	-1.23528	0.13596	0.04816

Supplementary Table 2. Cartesian coordinates of optimized geometries of four conformers of Criegee intermediates *syn-trans*-, *syn-cis*-, *anti-trans*-, and *anti-cis*-$C_2H_3C(CH_3)OO$ and dioxole predicted with the B3LYP/aug-cc-pVTZ method.

	x	*y*	*z*	*x*	*y*	*z*
	syn-trans- $C_2H_3C(CH_3)OO$			*syn-cis*-$C_2H_3C(CH_3)OO$		
C_1	-2.44486	-0.04447	0.00000	2.28191	-0.51004	0.00000
C_2	-1.25774	-0.66043	0.00000	1.38906	0.48547	0.00000
C_3	0.02300	0.00082	0.00000	-0.05196	0.32295	0.00000
C_4	0.28325	1.45131	0.00000	-1.00585	1.44766	0.00000
H_1	-2.54373	1.03155	0.00000	1.97958	-1.54785	0.00000
H_2	-3.36098	-0.61689	0.00000	3.34128	-0.29873	0.00000
H_3	-1.20522	-1.74235	0.00000	1.72886	1.51297	0.00000
H_4	0.90061	1.69853	0.86703	-1.66695	1.35621	-0.86597
H_5	0.90061	1.69853	-0.86703	-1.66695	1.35621	0.86597
H_6	-0.63139	2.03450	0.00000	-0.49474	2.40608	0.00000
O_1	1.01810	-0.80576	0.00000	-0.50065	-0.87065	0.00000
O_2	2.27167	-0.26765	0.00000	-1.86185	-1.03699	0.00000
	anti-trans-$C_2H_3C(CH_3)OO$			*anti-cis*-$C_2H_3C(CH_3)OO$		
C_1	-2.05395	-0.87958	0.00000	1.75463	1.03612	0.00000
C_2	-0.71361	-0.86605	0.00000	0.41492	1.15505	0.00000
C_3	0.05380	0.34580	0.00000	-0.56379	0.10647	0.00000
C_4	-0.51097	1.72181	0.00000	-2.02292	0.43163	0.00000
H_1	-2.64000	0.02836	0.00000	2.22852	0.07046	0.00000
H_2	-2.59672	-1.81245	0.00000	2.36166	1.93281	0.00000
H_3	-0.12962	-1.77360	0.00000	-0.02079	2.14761	0.00000
H_4	-1.13347	1.88406	-0.88005	-2.28002	1.02574	0.87933
H_5	-1.13347	1.88406	0.88005	-2.28002	1.02574	-0.87933
H_6	0.29635	2.44804	0.00000	-2.61539	-0.47916	0.00000
O_1	1.34510	0.30306	0.00000	-0.32435	-1.14998	0.00000
O_2	1.99034	-0.87682	0.00000	0.96298	-1.61236	0.00000
	dioxole					
C_1	-2.17104	0.06146	-0.00224			
C_2	-0.68504	0.12599	-0.00172			
C_3	0.16846	1.14150	-0.01503			
C_4	1.55813	0.58960	0.02299			
H_1	-0.08531	-1.09937	0.02460			
H_2	1.36535	-0.84247	-0.03678			
H_3	-2.53256	-0.46052	0.88493			
H_4	-2.58656	1.06607	-0.01377			
H_5	-2.53179	-0.48195	-0.87664			
H_6	-0.08485	2.18670	-0.02954			
O_1	2.09931	0.83606	0.94487			
O_2	2.17307	0.87700	-0.83639			

Supplementary Table 3. Cartesian coordinates of optimized geometries of nine conformers of iodoperoxy radical $C_2H_3C(CH_3)IOO$ predicted with the B3LYP/aug-cc-pVTZ-pp method.

	x	*y*	*z*	*x*	*y*	*z*
	**$C_2H_3C(CH_3)IOO$-1**			**$C_2H_3C(CH_3)IOO$-2**		
C_1	2.39257	-1.89435	-0.14418	2.41424	-1.74673	-0.51159
C_2	1.62653	-0.95586	-0.68620	1.51134	-1.28223	0.34421
C_3	0.97374	0.15469	0.05624	0.92411	0.08481	0.34802
C_4	1.39627	0.40961	1.48068	1.08023	0.83187	1.65475
H_1	2.60617	-1.93616	0.91454	2.79946	-1.15567	-1.32886
H_2	2.82620	-2.67034	-0.75872	2.79081	-2.75440	-0.40769
H_3	1.43329	-0.95550	-1.75188	1.14670	-1.91003	1.14748
H_4	1.18327	-0.45956	2.09701	0.57522	1.79160	1.62484
H_5	2.46892	0.61345	1.50515	0.66947	0.23444	2.46499
H_6	0.86535	1.26549	1.88460	2.14507	0.99206	1.83936
O_1	1.19603	1.32725	-0.78769	1.48731	0.80649	-0.76877
O_2	0.80771	2.46346	-0.25965	1.26733	2.10188	-0.74872
I_1	-1.24052	-0.23524	0.00469	-1.27818	-0.14700	-0.07948
	**$C_2H_3C(CH_3)IOO$-3**			**$C_2H_3C(CH_3)IOO$-4**		
C_1	2.42023	-1.57657	-0.79032	-2.09404	2.05561	-0.23337
C_2	1.68024	-0.47482	-0.81681	-1.45288	0.98093	-0.67437
C_3	0.93571	0.07554	0.34538	-0.84861	-0.07038	0.18443
C_4	1.23491	-0.48570	1.71493	-1.20903	-0.08962	1.65103
H_1	2.53924	-2.18424	0.09584	-2.23902	2.25996	0.81805
H_2	2.93128	-1.91350	-1.68119	-2.49535	2.77911	-0.92876
H_3	1.58039	0.10246	-1.72570	-1.32742	0.81709	-1.73759
H_4	0.97906	-1.54059	1.75953	-0.90779	0.83774	2.13170
H_5	2.29767	-0.36570	1.93261	-2.28846	-0.21052	1.74812
H_6	0.65888	0.04133	2.47131	-0.71205	-0.91681	2.15140
O_1	1.16582	1.51383	0.48278	-1.11908	-1.40373	-0.40570
O_2	1.05943	2.20140	-0.62909	-2.41620	-1.63141	-0.46285
I_1	-1.25311	-0.17155	-0.08304	1.35622	0.02746	-0.05285
	**$C_2H_3C(CH_3)IOO$-5**			**$C_2H_3C(CH_3)IOO$-6**		
C_1	2.63199	-0.32753	1.34212	-2.21329	1.86661	-0.13279
C_2	1.58025	0.43451	1.07061	-1.35573	1.19706	0.62693
C_3	0.84622	0.51248	-0.22332	-0.81042	-0.15792	0.34280
C_4	0.84234	1.89870	-0.84058	-0.98690	-1.15730	1.46646
H_1	3.05085	-1.02375	0.63081	-2.58662	1.47714	-1.06850
H_2	3.10404	-0.27200	2.31291	-2.56987	2.83900	0.17570
H_3	1.19438	1.11423	1.82078	-1.00151	1.62291	1.55827
H_4	0.25453	1.91526	-1.75514	-2.05006	-1.29497	1.65900
H_5	0.42496	2.61708	-0.13961	-0.51121	-0.77712	2.36771
H_6	1.87048	2.18744	-1.06764	-0.54090	-2.11528	1.20931
O_1	1.40540	-0.34583	-1.24590	-1.31447	-0.67671	-0.93398
O_2	1.35444	-1.63359	-0.98253	-2.51365	-1.21401	-0.80801

I_1	-1.27137	-0.10966	0.14967	1.36006	0.05440	-0.10917
	**$C_2H_3C(CH_3)IOO$-7**			**$C_2H_3C(CH_3)IOO$-8**		
C_1	-1.34558	2.21363	-0.48362	0.93963	-2.54732	-0.33936
C_2	-1.75602	1.00984	-0.12057	1.57755	-1.40093	-0.16830
C_3	-0.94824	-0.12268	0.43106	1.01944	-0.05363	0.17691
C_4	-1.09054	-0.28983	1.93461	1.43250	0.42365	1.55613
H_1	-0.30037	2.48890	-0.49447	-0.13699	-2.62340	-0.28365
H_2	-2.06028	2.96254	-0.79487	1.49325	-3.45327	-0.54332
H_3	-2.81580	0.77260	-0.14826	2.66147	-1.37674	-0.24328
H_4	-0.58863	-1.19407	2.27210	1.09754	1.44138	1.73165
H_5	-2.15054	-0.35776	2.18794	2.52194	0.39079	1.63190
H_6	-0.66345	0.56922	2.44450	1.00866	-0.23504	2.30973
O_1	-1.42679	-1.39037	-0.11580	1.57779	0.82542	-0.86329
O_2	-1.66091	-1.36151	-1.40884	1.45852	2.10763	-0.61295
I_1	1.20987	-0.00174	-0.07243	-1.18398	0.07286	-0.00274
	**$C_2H_3C(CH_3)IOO$-9**					
C_1	-0.85679	2.49716	-0.16648			
C_2	-1.47631	1.33291	-0.05901			
C_3	-0.89363	-0.01110	0.24765			
C_4	-1.24917	-0.52097	1.63195			
H_1	0.21643	2.59302	-0.07871			
H_2	-1.42190	3.40015	-0.35148			
H_3	-2.55621	1.28131	-0.16151			
H_4	-0.88415	-1.53528	1.77847			
H_5	-2.33304	-0.51140	1.74686			
H_6	-0.81049	0.13087	2.38376			
O_1	-1.39059	-0.97870	-0.77997			
O_2	-2.68859	-1.16469	-0.68266			
I_1	1.26940	-0.15094	-0.06681			

Supplementary Table 4. Cartesian coordinates of optimized geometries of six conformers of iodoperoxy radical $C(CH_3)ICHCH_2OO$ predicted with the B3LYP/aug-cc-pVTZ-pp method.

	x	*y*	*z*	*x*	*y*	*z*
	**(*Z*)-C(CH₃)ICHCH₂OO-1**			**(*Z*)-C(CH₃)ICHCH₂OO-2**		
C_1	-1.93990	-0.57964	0.47207	2.01366	-0.00210	0.56055
C_2	-1.32212	0.75917	0.27465	4.01900	-0.86918	-0.17093
C_3	-0.04772	1.06450	0.06597	-0.16532	1.19392	0.05182
C_4	0.48084	2.45228	-0.10650	-0.96368	2.43584	-0.18200
H_1	-1.25808	-1.39637	0.25046	1.49103	-0.95249	0.48647
H_2	-2.35891	-0.69584	1.47152	2.51845	0.06865	1.52411
H_3	-2.02274	1.58870	0.31726	1.64187	2.13308	0.31147
H_4	1.20142	2.69935	0.67429	-1.45991	2.40194	-1.15277
H_5	0.99309	2.55786	-1.06380	-1.74173	2.55109	0.57383
H_6	-0.33814	3.17163	-0.06623	-0.31359	3.31121	-0.15097
O_1	-3.05319	-0.76906	-0.46649	3.07480	-0.00079	-0.45979
O_2	-4.11955	-0.09243	-0.09691	4.01900	-0.86918	-0.17093
I_1	1.47432	-0.43795	-0.02478	-1.34028	-0.59120	-0.01699
	**(*Z*)-C(CH₃)ICHCH₂OO-3**			**(*E*)-C(CH₃)ICHCH₂OO-1**		
C_1	1.98740	-0.27280	-0.85219	-2.57328	0.03534	0.64762
C_2	1.29033	0.97901	-0.45186	-1.14017	-0.32932	0.45685
C_3	0.02760	1.14266	-0.07823	-0.17255	0.52586	0.13698
C_4	-0.58880	2.45343	0.29199	-0.26536	1.99478	-0.11752
H_1	1.41635	-1.16882	-0.62391	-2.76358	1.10444	0.67260
H_2	2.26209	-0.26390	-1.90828	-2.99397	-0.42833	1.53889
H_3	1.90503	1.87504	-0.48265	-0.90496	-1.37588	0.59529
H_4	-1.41382	2.70445	-0.37598	0.34686	2.54988	0.59444
H_5	-0.99183	2.42042	1.30511	0.10817	2.23150	-1.11436
H_6	0.15797	3.24684	0.23845	-1.29141	2.35219	-0.04467
O_1	3.28769	-0.38556	-0.18185	-3.33588	-0.50669	-0.49474
O_2	3.15351	-0.65805	1.09953	-4.63092	-0.37782	-0.30757
I_1	-1.34273	-0.49583	0.01976	1.81399	-0.23996	-0.04844
	**(*E*)-C(CH₃)ICHCH₂OO-2**			**(*E*)-C(CH₃)ICHCH₂OO-3**		
C_1	2.61026	-0.25949	-0.92265	-2.63033	0.51860	0.50438
C_2	1.17265	-0.50923	-0.60978	-1.24380	-0.02135	0.37574
C_3	0.28715	0.43389	-0.30052	-0.16399	0.69476	0.07926
C_4	0.48469	1.90915	-0.18388	-0.05107	2.15908	-0.19607
H_1	2.85268	0.78415	-1.09531	-2.70867	1.58317	0.30535
H_2	2.94133	-0.86217	-1.76776	-3.06718	0.28483	1.47452
H_3	0.86578	-1.54600	-0.63521	-1.16032	-1.08614	0.54748
H_4	-0.15880	2.44249	-0.88488	0.59284	2.64165	0.54054
H_5	0.21981	2.24708	0.81854	0.39760	2.33045	-1.17506
H_6	1.51946	2.19086	-0.36967	-1.02312	2.65012	-0.17494
O_1	3.48715	-0.72416	0.17047	-3.51283	-0.09460	-0.50017
O_2	3.47194	0.10625	1.18924	-3.89364	-1.30122	-0.13977
I_1	-1.72154	-0.18413	0.09731	1.71237	-0.32725	-0.01846

Supplementary Table 5. Comparison of experimental vibrational wavenumbers and IR intensities of (*Z*)-(CH_2I)HC=C(CH_3)I (1) with those of (*Z*)- and (*E*)-(CH_2I)HC=C(CH_3)I predicted with the B3LYP/aug-cc-pVTZ-pp method.

Mode	This work (gas)		*p*-H_2 [a]		(*Z*)-conformer		(*E*)-conformer	
	ν /cm^{-1}	Intensity[b]	ν /cm^{-1}	Intensity[b]	ν /cm^{-1}[c]	Intensity[d]	ν /cm^{-1}[c]	Intensity[d]
ν_1					3100	0.6	3109	1.1
ν_2	3010	8	2982.6	7	3035	6.7	3087	0.4
ν_3	2975	29			3031	2.1	3017	5.0
ν_4	2929	18	2972.4	18	3012	12.0	3023	12.1
ν_5	2888	4	2824.5	15	3000	5.9	3003	4.0
ν_6	2854	5	2847.9	5	2945	17.0	2953	9.4
ν_7	1641	17	1641.2	20	1644	33.5	1633	69.2
ν_8			1439.8	3	1453	3.5	1460	6.2
ν_9	1434	24	1432.0	29	1442	6.4	1440	4.6
ν_{10}			1430.3	18	1434	12.5	1435	11.2
ν_{11}	1384	3	1381.6	1	1383	2.1	1385	6.9
ν_{12}	1294	29	1299.1	41	1300	29.3	1339	2.2
ν_{13}	1169	[e]	1168.6	25	1157	30.8	1149	22.2
ν_{14}	1152	100	1153.0	100	1143	73.3	1134	120.9
ν_{15}			1095.0	<1	1084	1.3	1059	3.8
ν_{16}	1063	24	1061.6	31	1049	31.9	1052	39.9
ν_{17}			1038.2	<1	1043	1.2	1039	3.2
ν_{18}	965	2	965.3	1	957	2.7	917	4.7
ν_{19}	837	20	837.9	27	853	15.3	878	12.3
ν_{20}			810.6	8	806	8.6	825	1.2
ν_{21}			547.3	16	547	38.6	608	8.7
ν_{22}			530.3	10	519	11.4	509	24.3
ν_{23}					424	8.5	426	31.7
ν_{24}					410	9.9	353	11.5
ν_{25}					259	1.7	291	3.7
ν_{26}					264	3.0	257	1.2
ν_{27}					201	0.4	203	0.5

[a]Haupa et al. J. Phys. Chem. A (submitted). [b]Percentage IR intensities relative to the most intense band near 1152 cm^{-1}. [c]Harmonic vibrational wavenumber x scaled according to y = (0.9708 ±0.0159) x + (9.3 ± 20.7); see text. [d]In unit of km mol^{-1}. [e]overlapped with the band at 1152 cm^{-1}.

Supplementary Table 6. Vibrational wavenumbers and IR intensities of (*Z*)- and (*E*)-$C_2H_3CC(CH_3)I$ and (*Z*)- and (*E*)-$(CH_2I)CHC(CH_3)$ predicted with the B3LYP/aug-cc-pVTZ-pp method.

Mode	Symmetry	(*Z*)-$C_2H_3CC(CH_3)I$		(*E*)- $C_2H_3CC(CH_3)I$	
		ν /cm^{-1}[a]	Intensity[b]	ν /cm^{-1}[a]	Intensity[b]
ν_1	a′	3161	5.6	3158	6.8
ν_2	a′	3072	2.3	3076	2.8
ν_3	a′	3022	15.6	3072	4.9
ν_4	a′	3001	13.3	3019	10.4
ν_5	a′	2930	21.1	2939	14.1
ν_6	a′	1483	5.3	1487	0.6
ν_7	a′	1455	1.2	1453	0.1
ν_8	a′	1418	8.9	1410	23.4
ν_9	a′	1379	3.1	1374	7.5
ν_{10}	a′	1261	37.0	1311	0.7
ν_{11}	a′	1221	1.6	1214	5.6
ν_{12}	a′	1108	35.3	1088	76.1
ν_{13}	a′	1018	7.5	999	5.6
ν_{14}	a′	887	10.3	888	8.3
ν_{15}	a′	537	8.9	612	18.0
ν_{16}	a′	438	6.2	347	2.4
ν_{17}	a′	281	0.4	288	0.6
ν_{18}	a′	194	0.1	225	0.2
ν_{19}	a″	2973	9.1	2979	8.7
ν_{20}	a″	1429	11.0	1431	10.5
ν_{21}	a″	1013	1.6	1016	1.5
ν_{22}	a″	930	10.4	951	9.5
ν_{23}	a″	803	40.8	790	44.0
ν_{24}	a″	560	2.5	555	2.0
ν_{25}	a″	314	5.5	313	3.5
ν_{26}	a″	219	0.4	175	0.2
ν_{27}	a″	148	0.5	91	0.2

Mode	(*Z*)-(CH_2I)CHC(CH_3)		(*E*)-(CH_2I)CHC(CH_3)	
	ν /cm^{-1a}	Intensity^{b}	ν /cm^{-1a}	Intensity^{b}
v_1	3097	0.2	3093	0.6
v_2	3027	4.2	3026	2.8
v_3	2991	5.7	3025	3.5
v_4	2968	11.4	2990	5.3
v_5	2945	12.1	2967	11.1
v_6	2888	22.5	2889	16.8
v_7	1704	30.4	1696	25.2
v_8	1441	6.0	1443	8.2
v_9	1435	9.0	1430	9.0
v_{10}	1417	9.0	1419	7.2
v_{11}	1365	2.1	1365	4.9
v_{12}	1276	0.2	1295	1.4
v_{13}	1146	72.2	1145	67.0
v_{14}	1129	9.3	1110	9.8
v_{15}	1064	6.6	1031	0.1
v_{16}	1026	1.1	1022	0.6
v_{17}	1012	1.3	1007	4.5
v_{18}	902	7.3	884	6.8
v_{19}	812	5.7	821	11.6
v_{20}	768	19.7	779	38.7
v_{21}	509	38.0	533	41.2
v_{22}	430	1.8	461	0.2
v_{23}	250	10.4	364	15.9
v_{24}	244	3.1	204	0.4
v_{25}	203	6.4	191	3.6
v_{26}	134	1.9	118	0.0
v_{27}	80	0.2	62	0.6

[a]Harmonic vibrational wavenumber x scaled according to $y = (0.9708 \pm 0.0159)\,x + (9.3 \pm 20.7)$; see text. [b]In unit of km mol^{-1}.

Supplementary Table 7. Vibrational wavenumbers and IR intensities of four conformers of Criegee intermediates $C_2H_3C(CH_3)OO$ and dioxole predicted with the B3LYP/aug-cc-pVTZ method.

Mode	Symmetry	*syn-trans*-$C_2H_3C(CH_3)OO$			*syn-cis*-$C_2H_3C(CH_3)OO$		
		Harmonic[a]	Anharmonic	Intensity[b]	Harmonic[a]	Anharmonic	Intensity[b]
ν_1	a′	3155	3098	3.0	3159	3113	0.8
ν_2	a′	3086	3034	0.7	3090	3037	5.3
ν_3	a′	3076	3102	4.7	3072	3012	1.1
ν_4	a′	3070	2967	1.3	3058	3001	6.9
ν_5	a′	2954	2917	22.3	2946	2909	5.6
ν_6	a′	1626	1627	3.8	1630	1634	12.7
ν_7	a′	1455	1445	14.6	1460	1440	19.7
ν_8	a′	1454	1437	22.3	1452	1449	48.1
ν_9	a′	1425	1420	15.3	1403	1394	0.1
ν_{10}	a′	1367	1365	18.2	1366	1364	23.3
ν_{11}	a′	1304	1308	6.5	1308	1319	0.4
ν_{12}	a′	1278	1282	1.3	1244	1239	26.5
ν_{13}	a′	1045	1048	34.5	1084	1086	9.2
ν_{14}	a′	1000	1004	32.4	1011	1010	12.3
ν_{15}	a′	948	947	127.4	935	934	85.5
ν_{16}	a′	792	791	0.1	794	792	2.1
ν_{17}	a′	594	596	8.7	604	604	29.1
ν_{18}	a′	490	489	8.5	479	478	5.8
ν_{19}	a′	331	333	5.1	325	313	6.7
ν_{20}	a′	259	254	8.1	232	221	5.9
ν_{21}	a″	2990	2923	0.6	2982	2914	0.8
ν_{22}	a″	1409	1396	8.9	1408	1393	9.8
ν_{23}	a″	1020	1033	0.1	1019	1017	8.9
ν_{24}	a″	1004	951	16.9	999	947	12.4
ν_{25}	a″	948	939	43.6	971	968	33.9
ν_{26}	a″	676	666	5.6	659	647	7.7
ν_{27}	a″	459	448	1.1	445	435	1.2
ν_{28}	a″	284	267	0.2	271	272	1.4

Mode	Symmetry	Harmonic[a]	Anharmonic	Intensity[b]	Harmonic[a]	Anharmonic	Intensity[b]
ν_{29}	a″	193	143	2.1	195	162	0.5
ν_{30}	a″	126	100	1.4	78	32	0.2

		***anti-trans*-$C_2H_3C(CH_3)OO$**			***anti-cis*-$C_2H_3C(CH_3)OO$**		
**Mode**	**Symmetry**	**Harmonic[a]**	**Anharmonic**	**Intensity[b]**	**Harmonic[a]**	**Anharmonic**	**Intensity[b]**
ν_1	a′	3161	3120	2.3	3196	3128	1.9
ν_2	a′	3129	3074	8.8	3071	3017	13.2
ν_3	a′	3075	2988	1.7	3059	3092	4.8
ν_4	a′	3063	3001	2.3	3057	2922	6.6
ν_5	a′	2968	2945	7.8	2951	2910	4.7
ν_6	a′	1612	1617	4.7	1602	1594	28.5
ν_7	a′	1469	1465	1.3	1480	1471	12.5
ν_8	a′	1425	1427	38.5	1423	1432	50.9
ν_9	a′	1391	1385	20.9	1419	1419	21.3
ν_{10}	a′	1369	1360	3.6	1379	1373	3.0
ν_{11}	a′	1338	1333	15.9	1309	1313	6.4
ν_{12}	a′	1250	1246	0.5	1231	1229	22.7
ν_{13}	a′	1050	1055	124.6	1081	1093	4.2
ν_{14}	a′	999	1001	82.4	1014	1016	52.2
ν_{15}	a′	957	954	38.3	942	938	59.8
ν_{16}	a′	780	789	4.4	802	814	4.9
ν_{17}	a′	625	624	6.6	622	625	4.6
ν_{18}	a′	475	474	2.5	407	404	7.0
ν_{19}	a′	323	331	3.2	340	338	2.8
ν_{20}	a′	247	256	4.8	302	293	8.6
ν_{21}	a″	3017	2960	5.1	2996	2925	5.3
ν_{22}	a″	1439	1433	10.7	1441	1437	8.9
ν_{23}	a″	1029	1031	12.2	1028	1015	2.2
ν_{24}	a″	1025	1006	4.9	1010	991	7.1
ν_{25}	a″	962	960	38.4	999	1058	39.8
ν_{26}	a″	712	707	4.4	710	705	5.5
ν_{27}	a″	385	383	1.0	385	368	0.2
ν_{28}	a″	265	258	0.1	280	271	0.4
ν_{29}	a″	120	111	1.2	169	223	1.7
ν_{30}	a″	91	125	0.1	122	51	0.1

Mode	Symmetry	dioxole		
		Harmonic[a]	Anharmonic	Intensity[b]
ν_1	a	3171	3128	0.6
ν_2	a	3052	2989	8.0
ν_3	a	3011	2946	7.1
ν_4	a	2963	2938	16.9
ν_5	a	2927	2701	37.8
ν_6	a	2895	2894	78.5
ν_7	a	1700	1696	56.9
ν_8	a	1500	1495	3.4
ν_9	a	1462	1449	8.2
ν_{10}	a	1446	1434	7.7
ν_{11}	a	1392	1379	3.8
ν_{12}	a	1341	1335	4.4
ν_{13}	a	1266	1268	40.8
ν_{14}	a	1188	1178	20.0
ν_{15}	a	1153	1115	0.2
ν_{16}	a	1056	1045	0.7
ν_{17}	a	1038	1026	5.9
ν_{18}	a	1017	1028	9.8
ν_{19}	a	1011	1001	1.1
ν_{20}	a	953	952	31.2
ν_{21}	a	906	877	6.7
ν_{22}	a	839	829	13.4
ν_{23}	a	756	760	3.2
ν_{24}	a	708	697	31.0
ν_{25}	a	622	611	2.7
ν_{26}	a	564	554	0.0
ν_{27}	a	339	331	2.2
ν_{28}	a	237	262	5.1
ν_{29}	a	173	112	1.0
ν_{30}	a	60	-888	2.1

[a]Harmonic vibrational wavenumber x scaled according to $y = (0.9708 \pm 0.0159)\, x + (9.3 \pm 20.7)$; see text. [b]In unit of km mol^{-1}.

Supplementary Table 8. Vibrational wavenumbers and IR intensities of nine conformers of iodoperoxy radical $C_2H_3C(CH_3)IOO$ predicted with the B3LYP/aug-cc-pVTZ-pp method.

Mode	$C_2H_3C(CH_3)IOO$-1		$C_2H_3C(CH_3)IOO$-2		$C_2H_3C(CH_3)IOO$-3	
	ν [a]/cm^{-1}	Int.[b]	ν [a]/cm^{-1}	Int.[b]	ν [a]/cm^{-1}	Int.[b]
ν_1	3148	3.7	3157	1.8	3146	3.5
ν_2	3083	0.3	3084	1.0	3102	1.5
ν_3	3073	0.5	3073	0.1	3068	3.0
ν_4	3069	2.2	3071	3.1	3062	1.8
ν_5	3041	4.5	3034	4.4	3037	4.2
ν_6	2961	9.8	2956	9.1	2964	8.9
ν_7	1654	1.0	1647	1.2	1649	1.1
ν_8	1457	4.3	1455	4.9	1456	2.2
ν_9	1452	2.7	1442	2.1	1454	5.5
ν_{10}	1421	15.7	1422	13.2	1420	15.8
ν_{11}	1376	15.4	1376	13.6	1383	12.2
ν_{12}	1305	0.1	1304	0.1	1299	0.7
ν_{13}	1247	9.3	1205	14.3	1245	11.2
ν_{14}	1145	4.2	1170	14.0	1155	6.4
ν_{15}	1108	31.7	1103	10.2	1102	28.2
ν_{16}	1054	56.8	1050	74.7	1054	50.9
ν_{17}	1008	13.4	1020	10.0	1017	14.1
ν_{18}	985	3.6	1002	8.2	985	4.1
ν_{19}	954	39.3	956	37.3	953	38.6
ν_{20}	847	11.4	870	7.3	851	11.0
ν_{21}	767	69.8	754	61.0	735	75.2
ν_{22}	680	12.0	686	26.3	701	6.1
ν_{23}	569	4.7	569	6.8	594	9.7
ν_{24}	484	27.8	522	19.6	462	20.1
ν_{25}	416	8.3	413	10.9	423	8.2
ν_{26}	332	2.5	329	4.0	332	0.8
ν_{27}	286	0.6	268	0.3	268	2.3
ν_{28}	261	0.5	259	0.4	258	0.8
ν_{29}	249	4.9	243	6.9	246	6.4
ν_{30}	219	4.5	222	2.7	230	1.8
ν_{31}	213	1.0	207	0.3	215	1.8
ν_{32}	102	0.1	95	0.4	104	0.2
ν_{33}	89	0.2	70	0.1	96	0.1

Mode	$C_2H_3C(CH_3)IOO$-4		$C_2H_3C(CH_3)IOO$-5		$C_2H_3C(CH_3)IOO$-6	
	ν [a]/cm^{-1}	Int.[b]	ν [a]/cm^{-1}	Int.[b]	ν [a]/cm^{-1}	Int.[b]
ν_1	3148	3.9	3154	2.0	3155	1.7
ν_2	3083	0.2	3079	0.9	3079	1.5
ν_3	3069	2.9	3069	1.9	3069	2.1
ν_4	3057	3.0	3058	3.3	3053	4.0
ν_5	3039	2.7	3032	4.6	3041	2.2
ν_6	2969	4.0	2960	9.1	2973	3.4
ν_7	1652	1.0	1648	0.9	1648	0.4
ν_8	1455	2.6	1453	6.1	1455	6.7
ν_9	1455	4.7	1447	2.8	1445	1.8
ν_{10}	1419	16.0	1423	14.4	1421	14.2
ν_{11}	1375	11.1	1381	10.9	1375	13.2
ν_{12}	1306	0.3	1303	0.1	1301	0.0
ν_{13}	1212	9.4	1199	14.5	1183	10.8
ν_{14}	1131	15.5	1137	10.5	1129	8.7
ν_{15}	1098	20.5	1104	13.9	1085	8.9
ν_{16}	1059	83.9	1061	79.9	1067	107.5
ν_{17}	1005	11.2	1019	7.8	1015	3.8
ν_{18}	983	4.5	995	6.8	999	9.4
ν_{19}	952	38.0	953	38.7	956	33.8
ν_{20}	881	17.9	872	7.6	885	9.6
ν_{21}	740	74.8	754	58.4	715	66.8
ν_{22}	712	1.4	681	22.4	711	17.0
ν_{23}	538	9.3	615	21.1	626	10.6
ν_{24}	522	9.0	514	6.9	491	3.3
ν_{25}	394	3.1	365	10.0	378	3.6
ν_{26}	324	4.4	357	1.2	341	3.5
ν_{27}	288	1.9	268	0.9	280	0.7
ν_{28}	259	1.4	251	2.3	253	1.5
ν_{29}	249	2.7	246	3.8	247	3.8
ν_{30}	223	2.3	235	0.9	222	2.2
ν_{31}	181	2.2	194	1.1	192	0.8
ν_{32}	98	0.2	132	0.1	106	0.2
ν_{33}	85	0.5	101	0.4	76	0.9

Mode	$C_2H_3C(CH_3)IOO$-7		$C_2H_3C(CH_3)IOO$-8		$C_2H_3C(CH_3)IOO$-9	
	ν [a]/cm^{-1}	Int.[b]	ν [a]/cm^{-1}	Int.[b]	ν [a]/cm^{-1}	Int.[b]
ν_1	3147	0.4	3148	3.2	3145	3.7
ν_2	3067	1.8	3069	0.4	3065	1.9
ν_3	3061	3.1	3067	3.2	3053	4.1
ν_4	3045	3.3	3040	6.1	3051	0.3
ν_5	3029	5.9	3035	3.3	3036	3.8
ν_6	2958	11.0	2956	12.5	2968	4.3
ν_7	1669	6.9	1670	6.2	1665	7.9
ν_8	1452	4.7	1456	3.4	1455	4.5
ν_9	1447	1.8	1443	1.6	1445	2.1
ν_{10}	1416	21.2	1417	21.9	1416	17.5
ν_{11}	1380	9.8	1373	13.0	1373	10.7
ν_{12}	1301	4.0	1302	4.9	1292	5.0
ν_{13}	1192	21.5	1192	37.1	1167	53.8
ν_{14}	1146	15.4	1143	3.0	1129	4.7
ν_{15}	1082	44.8	1085	46.6	1090	56.2
ν_{16}	1060	30.8	1051	22.6	1040	14.7
ν_{17}	1024	6.6	1023	10.1	1019	8.1
ν_{18}	987	3.4	993	4.7	999	6.2
ν_{19}	962	36.9	970	38.5	963	36.0
ν_{20}	862	11.7	854	13.2	876	11.5
ν_{21}	749	42.2	756	33.7	725	27.3
ν_{22}	689	13.0	657	13.4	676	5.4
ν_{23}	568	14.9	595	11.9	632	21.3
ν_{24}	501	4.1	485	12.1	435	6.5
ν_{25}	419	14.9	397	11.7	377	2.8
ν_{26}	323	1.2	327	4.1	361	1.0
ν_{27}	285	3.5	316	0.4	307	4.4
ν_{28}	256	1.7	265	0.1	264	1.2
ν_{29}	255	1.5	260	2.2	251	0.1
ν_{30}	232	0.5	220	0.7	193	1.1
ν_{31}	177	0.6	186	0.4	187	0.4
ν_{32}	91	0.4	88	0.3	110	0.8
ν_{33}	72	0.4	66	0.3	80	0.3

[a]Harmonic vibrational wavenumber x scaled according to $y = (0.9708 \pm 0.0159)\,x + (9.3 \pm 20.7)$; see text. [b]In unit of km mol^{-1}.

Supplementary Table 9. Vibrational wavenumbers and IR intensities of six conformers of iodoperoxy radical $C(CH_3)ICHCH_2OO$ predicted with the B3LYP/aug-cc-pVTZ-pp method.

Mode	(*Z*)-$C(CH_3)ICHCH_2OO$-1		(*Z*)-$C(CH_3)ICHCH_2OO$-2		(*Z*)-$C(CH_3)ICHCH_2OO$-3	
	ν [a]/cm^{-1}	Int.[b]	ν [a]/cm^{-1}	Int.[b]	ν [a]/cm^{-1}	Int.[b]
ν_1	3054	2.0	3045	6.5	3046	4.5
ν_2	3040	1.4	3032	1.7	3033	2.5
ν_3	3015	12.1	3016	11.3	3015	11.7
ν_4	3005	5.0	3006	4.6	3005	5.0
ν_5	2980	8.6	2978	6.5	2973	11.5
ν_6	2948	13.0	2948	12.8	2948	13.8
ν_7	1668	24.7	1668	32.9	1669	23.6
ν_8	1453	4.1	1453	4.6	1453	4.4
ν_9	1439	4.8	1450	6.1	1439	5.4
ν_{10}	1435	12.9	1434	12.8	1435	12.4
ν_{11}	1384	3.0	1384	3.5	1384	3.0
ν_{12}	1337	4.6	1343	18.5	1338	7.4
ν_{13}	1272	44.4	1282	42.3	1280	40.8
ν_{14}	1239	7.0	1196	10.9	1234	9.4
ν_{15}	1137	38.1	1154	9.2	1127	21.4
ν_{16}	1113	23.7	1114	39.8	1106	34.0
ν_{17}	1057	8.5	1065	17.9	1061	7.6
ν_{18}	1045	0.9	1045	0.6	1045	0.6
ν_{19}	1015	11.4	1003	3.6	1012	6.0
ν_{20}	936	23.8	951	22.8	948	13.5
ν_{21}	837	21.6	862	16.0	860	35.9
ν_{22}	820	13.7	811	9.7	796	8.5
ν_{23}	563	2.3	582	35.5	566	6.6
ν_{24}	517	18.7	462	2.8	506	12.6
ν_{25}	428	6.9	427	8.8	432	10.5
ν_{26}	421	3.2	407	1.2	419	3.6
ν_{27}	347	3.9	344	4.9	330	5.1
ν_{28}	276	0.7	275	1.1	275	1.0
ν_{29}	214	0.4	209	0.4	217	1.0
ν_{30}	164	2.3	184	1.6	185	1.4
ν_{31}	144	0.4	122	1.4	145	1.6
ν_{32}	82	3.2	66	0.1	75	0.9
ν_{33}	44	3.2	41	2.4	34	2.1

Mode	(E)-C(CH3)ICHCH2OO-1		(E)-C(CH3)ICHCH2OO-2		(E)-C(CH3)ICHCH2OO-3	
	ν [a]/cm^{-1}	Int.[b]	ν [a]/cm^{-1}	Int.[b]	ν [a]/cm^{-1}	Int.[b]
ν_1	3093	0.6	3093	0.9	3094	0.4
ν_2	3049	10.2	3059	7.1	3058	8.7
ν_3	3027	12.8	3032	12.0	3024	15.5
ν_4	3008	3.3	3008	3.6	3007	3.8
ν_5	2988	9.0	2992	17.0	2988	13.2
ν_6	2957	7.2	2958	7.0	2956	8.9
ν_7	1652	60.3	1650	52.1	1654	43.9
ν_8	1464	6.1	1459	7.9	1456	7.7
ν_9	1443	4.4	1438	4.2	1438	10.4
ν_{10}	1436	11.7	1434	10.5	1435	2.8
ν_{11}	1385	6.4	1386	6.1	1386	6.5
ν_{12}	1351	27.4	1346	10.5	1345	9.0
ν_{13}	1314	26.3	1312	14.4	1303	8.6
ν_{14}	1195	26.4	1239	15.3	1244	22.8
ν_{15}	1149	8.9	1126	25.2	1136	29.4
ν_{16}	1102	70.7	1106	59.9	1102	60.5
ν_{17}	1061	14.6	1053	4.2	1053	7.5
ν_{18}	1042	0.6	1042	0.6	1041	0.7
ν_{19}	966	5.1	984	12.4	981	8.5
ν_{20}	931	17.4	910	7.5	917	9.2
ν_{21}	875	17.8	872	26.0	856	14.8
ν_{22}	829	6.1	794	15.7	825	17.2
ν_{23}	610	10.6	612	16.9	609	19.9
ν_{24}	515	35.1	544	2.8	541	0.6
ν_{25}	447	1.8	442	11.9	440	9.5
ν_{26}	350	5.0	371	10.6	391	7.7
ν_{27}	305	0.6	318	1.7	290	1.6
ν_{28}	279	2.0	286	2.2	274	1.6
ν_{29}	211	0.2	213	0.1	215	0.9
ν_{30}	180	0.3	179	0.2	182	0.2
ν_{31}	90	1.9	130	0.4	121	0.4
ν_{32}	70	0.0	64	1.7	67	2.5
ν_{33}	43	3.1	50	2.6	53	1.7

[a]Harmonic vibrational wavenumber x scaled according to y = (0.9708 ± 0.0159) x + (9.3 ± 20.7); see text. [b]In unit of km mol^{-1}.

Supplementary Table 10. Rotational parameters and ratios of types for each vibrational state of four conformers of Criegee intermediates MKVO predicted with the B3LYP/aug-cc-pVTZ method.

ν_i	Sym.	*syn-trans*-$C_2H_3C(CH_3)OO$				*syn-cis*-$C_2H_3C(CH_3)OO$			
		A'/*A*"	*B*'/*B*"	*C*'/*C*"	type ratio	*A*'/*A*"	*B*'/*B*"	*C*'/*C*"	type ratio
		A"=0.2887 cm^{-1}, B"=0.0785 cm^{-1}, C"=0.0624 cm^{-1}				*A*"=0.2171 cm^{-1}, B"=0.0916 cm^{-1}, C"=0.0652 cm^{-1}			
ν_1	a′	0.99950	0.99946	0.99950	*a*/*b* = 7/93	0.99986	0.99933	0.99952	*a*/*b* = 86/14
ν_2	a′	0.99930	0.99940	0.99939	*a*/*b* = 98/2	0.99980	0.99894	0.99922	*a*/*b* = 25/75
ν_3	a′	0.99913	0.99952	0.99944	*a*/*b* = 11/89	0.99990	0.99938	0.99952	*a*/*b* = 18/82
ν_4	a′	0.99909	0.99966	0.99957	*a*/*b* = 37/63	0.99945	0.99999	0.99991	*a*/*b* = 1/99
ν_5	a′	0.99998	0.99969	0.99989	*a*/*b* = 4/96	1.00016	0.99981	1.00006	*a*/*b* = 65/35
ν_6	a′	1.00016	0.99822	0.99848	*a*/*b* = 71/29	0.99852	0.99918	0.99890	*a*/*b* = 64/36
ν_7	a′	0.99972	0.99964	0.99960	*a*/*b* = 2/98	0.99859	0.99936	0.99917	*a*/*b* = 96/4
ν_8	a′	0.99797	0.99857	0.99893	*a*/b = 99/1	0.99994	0.99973	0.99948	*a*/*b* = 33/67
ν_9	a′	1.00039	0.99924	0.99917	*a*/b = 27/73	0.99938	0.99960	1.00011	*a*/*b* = 66/4
ν_{10}	a′	0.99128	0.99982	0.99923	*a*/*b* = 1/99	0.99483	0.99936	0.99868	*a*/*b* = 76/24
ν_{11}	a′	0.99955	0.99859	0.99817	*a*/*b* = 41/59	0.99966	1.00025	0.99965	*a*/*b* = 60/40
ν_{12}	a′	0.99972	1.00023	0.99891	*a*/*b* = 97/3	0.99930	0.99850	0.99765	*a*/*b* = 52/48
ν_{13}	a′	1.00153	1.00087	0.99958	*a*/*b* = 89/11	1.00174	0.99998	0.99911	*a*/*b* = 31/69
ν_{14}	a′	0.99851	0.99864	0.99925	*a*/*b* = 88/12	0.99933	0.99977	0.99945	*a*/ *b*= 93/7
ν_{15}	a′	1.00075	0.99795	0.99812	*a*/*b* = 95/5	1.00008	0.99698	0.99807	*a*/*b* = 63/37
ν_{16}	a′	0.99779	0.99940	0.99862	*a*/*b* = 99/1	0.99912	0.99895	0.99844	*a*/*b* = 84/16
ν_{17}	a′	1.00076	0.99943	0.99926	*a*/*b* = 99/1	0.99857	0.99926	0.99910	*a*/*b* = 90/10
ν_{18}	a′	1.00058	0.99957	0.99963	*a*/*b* = 43/5	1.00187	0.99984	0.99954	*a*/*b* = 99/1
ν_{19}	a′	0.99927	0.99987	0.99865	*a*/*b* = 42/58	0.99755	1.00223	0.99952	*a*/*b* = 1/99
ν_{20}	a′	1.00039	0.99918	0.99869	*a*/*b* = 90/10	0.99860	0.99969	0.99842	*a*/*b* = 74/26
ν_{21}	a″	0.99909	0.99966	0.99957	*c*	1.00091	0.99973	1.00014	*c*
ν_{22}	a″	1.00878	0.99964	1.00024	*c*	1.00587	0.99975	1.00026	*c*
ν_{23}	a″	1.00070	1.00069	0.99997	*c*	0.99898	0.99953	0.99997	*c*
ν_{24}	a″	0.99883	0.99864	1.00018	*c*	0.99865	0.99981	1.00029	*c*
ν_{25}	a″	0.99682	0.99944	1.00018	*c*	0.99891	0.99928	1.00002	*c*
ν_{26}	a″	1.00063	0.99996	1.00022	*c*	1.00070	1.00002	1.00041	*c*
ν_{27}	a″	0.99781	1.00023	1.00054	*c*	0.99816	1.00063	1.00086	*c*
ν_{28}	a″	1.00319	1.00070	1.00056	*c*	0.99974	1.00026	1.00043	*c*
ν_{29}	a″	0.99495	0.99929	0.99902	*c*	0.99279	1.00110	0.99903	*c*

ν_{30}	a″	0.99280	1.00088	1.00181	*c*	0.99785	0.99987	1.00317	*c*

ν_i	Sym.	***anti-trans*-$C_2H_3C(CH_3)OO$**				***anti-cis*-$C_2H_3C(CH_3)OO$**			
		***A*'/*A*"**	***B*'/*B*"**	***C*'/*C*"**	**type ratio**	***A*'/*A*"**	***B*'/*B*"**	***C*'/*C*"**	**type ratio**
		A"=0.1841 cm^{-1}, *B*"=0.0991 cm^{-1},*C*"=0.0652 cm^{-1}				*A*"=0.1582 cm^{-1}, *B*"=0.1214 cm^{-1}, *C*"=0.0696 cm^{-1}			
ν_1	a′	0.99904	0.99988	0.99962	*a*/*b* = 8/92	0.99695	1.00052	0.99899	*a*/*b* = 13/87
ν_2	a′	0.99910	0.99888	0.99897	*a*/*b* = 75/25	0.99958	0.99958	0.99960	*a*/*b* = 2/92
ν_3	a′	0.99875	1.00012	0.99963	*a*/*b* = 93/7	1.00002	0.99953	0.99976	*a*/*b* = 99/1
ν_4	a′	0.99930	1.00015	0.99991	*a*/*b* = 50/50	1.00004	0.99965	0.99987	*a*/*b* = 77/23
ν_5	a′	0.99965	0.99985	0.99992	*a*/*b* = 67/33	0.99948	1.00002	0.99996	*a*/*b* = 48/52
ν_6	a′	1.00081	0.99765	0.99857	*a*/*b* = 2/98	1.00094	0.99783	0.99915	*a*/*b* = 86/14
ν_7	a′	0.99906	1.00031	0.99992	*a*/*b* = 13/87	0.99894	0.99864	0.99898	*a*/*b* = 99/1
ν_8	a′	0.99991	0.99967	1.00061	*a*/*b* = 62/38	0.99921	0.99944	1.00060	*a*/*b* = 74/26
ν_9	a′	0.99566	0.99930	1.00049	*a*/b = 1/99	0.99970	1.00026	0.99970	*a*/*b* = 99/1
ν_{10}	a′	0.99812	0.99790	0.99565	*a*/*b* = 39/61	0.99980	0.99671	0.99766	*a*/*b* = 64/36
ν_{11}	a′	0.99778	0.99904	0.99755	*a*/*b* = 27/73	1.00138	0.99962	0.99999	*a*/*b* = 97/3
ν_{12}	a′	1.00089	1.00045	0.99966	*a*/*b* = 5/95	0.99863	0.99812	0.99731	*a*/*b* = 67/33
ν_{13}	a′	0.99930	1.00188	0.99933	*a*/*b* = 96/4	1.00153	1.00116	0.99945	*a*/*b* = 92/8
ν_{14}	a′	0.99945	0.99673	0.99914	*a*/*b* = 91/9	0.99944	0.99876	0.99892	*a*/*b* = 20/80
ν_{15}	a′	0.99923	0.99924	0.99903	*a*/*b* = 98/2	0.99872	0.99874	0.99840	*a*/*b* = 1/99
ν_{16}	a′	0.99782	0.99961	0.99844	*a*/*b* = 96/4	0.99913	0.99942	0.99850	*a*/*b* = 9/91
ν_{17}	a′	0.99979	0.99941	0.99920	*a*/*b* = 59/41	0.99922	0.99910	0.99911	*a*/*b* = 72/28
ν_{18}	a′	1.00123	0.99981	0.99965	*a*/*b* = 44/56	1.00025	1.00152	1.00082	*a*/*b* = 27/73
ν_{19}	a′	1.00128	1.00161	0.99945	*a*/*b* = 9/91	1.00561	1.00069	0.99957	*a*/*b* = 91/9
ν_{20}	a′	1.00220	0.99995	0.99807	*a*/b = 52/48	0.99666	1.00169	0.99645	*a*/*b* = 11/89
ν_{21}	a″	0.99987	0.99987	0.99994	*c*	0.99946	1.00026	0.99999	*c*
ν_{22}	a″	1.00344	0.99967	1.00011	*c*	0.99985	1.00264	1.00013	*c*
ν_{23}	a″	1.00060	1.00068	0.99997	*c*	0.99957	0.99894	0.99974	*c*
ν_{24}	a″	0.99914	0.99934	0.99979	*c*	0.99831	0.99907	0.99991	*c*
ν_{25}	a″	0.99749	0.99901	0.99992	*c*	1.00013	0.99792	1.00032	*c*
ν_{26}	a″	0.99991	0.99939	1.00015	*c*	0.99949	0.99961	1.00026	*c*
ν_{27}	a″	0.99896	1.00089	1.00106	*c*	1.00348	0.99808	1.00157	*c*
ν_{28}	a″	0.99839	0.99794	1.00071	*c*	0.99461	0.99943	1.00069	*c*
ν_{29}	a″	0.99079	1.00483	1.00274	*c*	0.99619	1.00094	1.00129	*c*
ν_{30}	a″	0.99308	1.00212	0.99946	*c*	1.00128	0.99873	1.00012	*c*

Supplementary Table 11. Comparison of observed vibrational wavenumbers of (*Z*)-$C_2H_3C(CH_3)I$ (2) in region 800–1450 cm^{-1} with those calculated with the B3LYP/aug-cc-pVTZ-pp method.

Mode	Sym.	Experiment		Calculation[a]		Mode description[d]
		ν /cm^{-1}	Intensity[b]	ν /cm^{-1}	Intensity[c]	
ν_8	a′	1406	13	1418	8.9	$C^{(2)}$H bend/$C^{(2)}C^{(3)}$ str.
ν_9	a′			1379	3.1	$C^{(4)}H_3$ umbrella
ν_{10}	a′	1261	38	1261	37.0	$C^{(2)}C^{(3)}$ str./$C^{(2)}$H bend
ν_{11}	a′			1221	1.6	$C^{(1)}C^{(2)}$ str.
ν_{12}	a′	1109	100	1108	35.3	$C^{(3)}C^{(4)}$ str./$C^{(1)}C^{(2)}C^{(3)}$ bend
ν_{13}	a′	1019	22	1018	7.5	CH *ip* bend/$C^{(4)}H_2$ wag
ν_{14}	a′	873	16	887	10.3	$C^{(1)}H_2$ *ip* bend/$C^{(3)}$I str.
ν_{20}	a″			1429	11.0	$C^{(4)}H_2$ twist
ν_{21}	a″			1013	1.6	$C^{(4)}H_2$ rock/$C^{(2)}C^{(3)}C^{(4)}$ *oop* def.
ν_{22}	a″	925	8	930	10.4	$C^{(2)}$H *oop* bend
ν_{23}	a″			803	40.8	$C^{(1)}H_2$ wag

[a]Harmonic vibrational wavenumber *x* scaled according to (0.9708 ± 0.0159) *x* + (9.3 ± 20.7); see text. [b]Percentage IR intensities relative to the most intense band near 1109 cm^{-1}. [c]In unit of km mol^{-1}. [d]Approximate mode description. str.: stretch; def.: deform; *ip*: in-plane; *oop*: out-of-plane.

Supplementary Table 12. Comparison of observed vibrational wavenumbers of *syn-trans*-$C_2H_3C(CH_3)OO$ (3) in region 800–1500 cm^{-1} with those calculated with the B3LYP/aug-cc-pVTZ method.

Mode	Sym.	Experiment		Harmonic[a]		Anharmonic	Mode description[d]
		ν /cm^{-1}	Int.[b]	ν /cm^{-1}	Int.[c]	ν /cm^{-1}	
ν_7	a′			1455	14.6	1445	asym. C$^{(2)}$C$^{(3)}$C$^{(4)}$ str.
ν_8	a′			1454	22.3	1437	C$^{(3)}$O str.
ν_9	a′	1416	12	1425	15.3	1420	C$^{(1)}$H$_2$ scissor
ν_{10}	a′	1346	7	1367	18.2	1365	C$^{(4)}$H$_3$ umbrella
ν_{11}	a′			1304	6.5	1308	C$^{(2)}$C$^{(3)}$ str./HC$^{(1)}$C$^{(2)}$ bend
ν_{12}	a′			1278	1.3	1282	C$^{(2)}$H *ip* bend
ν_{13}	a′	1060	45	1045	34.5	1048	CH *ip* bend/C$^{(4)}$H$_3$ wag
ν_{14}	a′	987	44	1000	32.4	1004	CH *ip* bend
ν_{15}	a′	948	100	948	127.4	947	OO str.
ν_{16}	a′			792	0.1	791	sym. C$^{(2)}$C$^{(3)}$C$^{(4)}$ str.
ν_{22}	a″	1383	10	1409	8.9	1396	C$^{(4)}$H$_3$ def.
ν_{23}	a″			1020	0.1	1033	*oop* def.
ν_{24}	a″			1004	16.9	951	C$^{(2)}$H *oop* bend
ν_{25}	a″	916/908	15	948	43.6	939	C$^{(1)}$H$_2$ wag

[a]Harmonic vibrational wavenumber x scaled according to (0.9708 ± 0.0159) x + (9.3 ± 20.7); see text. [b]Percentage IR intensities relative to the most intense band near 948 cm^{-1}. [c]In unit of km mol^{-1}. [d]Approximate mode description. str.: stretch; def.: deform; *ip*: in-plane; *oop*: out-of-plane.

Supplementary Table 13. Comparison of observed vibrational wavenumbers of *syn-cis*-$C_2H_3C(CH_3)OO$ in region 800–1500 cm^{-1} with those calculated with the B3LYP/aug-cc-pVTZ method.

Mode	Sym.	Experiment	Harmonic[a]		Anharmonic	Mode description[c]
		ν /cm^{-1}	ν /cm^{-1}	Int.[b]	ν /cm^{-1}	
ν_7	a′		1460	19.7	1440	$C^{(3)}O$ str.
ν_8	a′	[d]	1452	48.1	1449	asym. $C^{(2)}C^{(3)}C^{(4)}$ str.
ν_9	a′		1403	0.1	1394	$C^{(1)}H_2$ scissor/ $C^{(4)}H_3$ umbrella
ν_{10}	a′	[d]	1366	23.3	1364	$C^{(4)}H_3$ umbrella
ν_{11}	a′		1308	0.4	1319	$C^{(1)}H/C^{(2)}H$ *iph*. bend
ν_{12}	a′	1243	1244	26.5	1239	$C^{(2)}C^{(3)}$ str./$C^{(2)}H$ *ip* bend
ν_{13}	a′		1084	9.2	1086	CH *ip* bend
ν_{14}	a′		1011	12.3	1010	$C^{(4)}H_3$ wag/CH *ip* bend
ν_{15}	a′	[d]	935	85.5	934	OO str.
ν_{16}	a′		794	2.1	792	sym. $C^{(2)}C^{(3)}C^{(4)}$ str.
ν_{22}	a″		1408	9.8	1393	$C^{(4)}H_3$ def.
ν_{23}	a″	1031	1019	8.9	1017	*oop* def.
ν_{24}	a″		999	12.4	947	$C^{(2)}H$ *oop* bend
ν_{25}	a″	980	971	33.9	968	$C^{(1)}H_2$ wag

[a]Harmonic vibrational wavenumber *x* scaled according to (0.9708 ± 0.0159) *x* + (9.3 ± 20.7); see text. [b]In unit of km mol^{-1}. [c]Approximate mode description. str.: stretch; def.: deform; *ip*: in-plane; *oop*: out-of-plane; *iph*: in-phase. [d]Overlapped with *syn-trans*-MVKO.

Supplementary Table 14. Comparison of observed vibrational wavenumbers of $C_2H_3C(CH_3)IOO$ (4) in region 800–1500 cm^{-1} with $C_2H_3C(CH_3)IOO$-1 and $C_2H_3C(CH_3)IOO$-2 calculated with the B3LYP/aug-cc-pVTZ-pp method.

Mode	Experiment		$C_2H_3C(CH_3)IOO$-1[a]		$C_2H_3C(CH_3)IOO$-2[a]		Mode description[d]
	ν /cm^{-1}	Intensity[b]	ν /cm^{-1}	Intensity[c]	ν /cm^{-1}	Intensity[c]	
ν_8			1457	4.3	1455	4.9	$C^{(4)}H_3$ def.
ν_9			1452	2.7	1442	2.1	$C^{(4)}H_3$ def.
ν_{10}			1421	15.7	1422	13.2	$C^{(1)}H_2$ scissor
ν_{11}	1375	53	1376	15.4	1376	13.6	$C^{(4)}H_3$ umbrella
ν_{12}			1305	0.1	1304	0.1	$HC^{(2)}C^{(1)}$ bend
ν_{13}	1213	53	1247	9.3	1205	14.3	$C^{(2)}C^{(3)}$ str.
ν_{14}			1145	4.2	1170	14.0	OO str.
ν_{15}	1108	100	1108	31.7	1103	10.2	CH *ip* bend/$C^{(4)}H_2$ wag
ν_{16}	1063	80	1054	56.8	1050	74.7	$C^{(3)}C^{(4)}$ str./$C^{(4)}H_2$ wag
ν_{17}			1008	13.4	1020	10.0	$C^{(2)}H$ *oop* bend
ν_{18}	986	47	985	3.6	1002	8.2	CH *ip* bend/$C^{(4)}H_2$ wag
ν_{19}			954	39.3	956	37.3	$C^{(1)}H_2$ wag
ν_{20}	885	100	847	11.4	870	7.3	$C^{(2)}C^{(3)}$ str.

[a]Harmonic vibrational wavenumber *x* scaled according to (0.9708 ± 0.0159) *x* + (9.3 ± 20.7); see text. [b]Percentage IR intensities relative to the most intense band near 1109 cm^{-1}. [c]In unit of km mol^{-1}. [d]Approximate mode description. str.: stretch; def.: deform; *ip*: in-plane; *oop*: out-of-plane.

Supplementary Table 15. Summary on estimates of concentrations of species in varied experiments.

	Description	Unit	Expt. 1	Expt. 2	Expt. 3	Expt. 4
1	Pressure of (*Z*)-(CH_2I)HC=C(CH_3)I **(1)**	mTorr	40	40	35	42
2	Pressure of O_2	Torr	35	82	246	347
3	Probed period	µs	0–5	0–5	0–5	12.5–25
4	Integrated absorbance 1130–1190 cm^{-1} for **(1)**	cm^{-1}	0.35 ± 0.02[a]	0.33 ± 0.02	0.11 ± 0.01	0.081 ± 0.004
5	Integrated absorbance, B_6 (920–960 cm^{-1}) for **(3)**	cm^{-1}	0.12 ± 0.01	0.10 ± 0.01	0.028 ± 0.002	0.017 ± 0.001
6	Integrated absorbance, C_3 (1100–1120 cm^{-1}) for **(4)**	cm^{-1}	0.021 ± 0.002	0.019 ± 0.002	0.011 ± 0.001	0.009 ± 0.001
7	Integrated absorbance, C_6 (862–907 cm^{-1}) for **(4)**	cm^{-1}	0.027 ± 0.003	0.025 ± 0.003	0.015 ± 0.002	0.018[b] ± 0.002
8	Loss of **(1)** from raw 4 upon irradiation	10^{13} [c]	34 ± 2[d]	32 ± 2	11 ± 1	7.9 ± 0.4
9	**(3)** after irradiation, from row 5	10^{13} [c]	9.6 ± 1.0	8.0 ± 0.8	2.2 ± 0.2	1.4 ± 0.1
10	**(4)** after irradiation, from row 6	10^{13} [c]	10.5 ± 1.0	9.5 ± 1.0	5.5 ± 0.6	4.5 ± 0.5
11	**(4)** from after irradiation, row 7	10^{13} [c]	15.4 ± 1.7	14.3 ± 1.7	8.6 ± 1.1	10.3[b] ± 1.1
12	**(3)** from rows 8 & 9, relative to experiment 1		1.00	0.89 ± 0.15[e]	0.71 ± 0.12	0.63 ± 0.09
13	**(4)** from rows 8 & 10, relative to experiment 1		1.00	0.96 ± 0.16	1.62 ± 0.29	1.84 ± 0.30
14	**(4)** from rows 8 & 11s, relative to experiment 1		1.00	0.97 ± 0.18	1.73 ± 0.35	2.87[b] ± 0.49

[a]Error bars reflect mainly uncertainties in baseline. [b]Might be interfered by an unknown band appeared at high pressure. [c]In unit of molecule cm^{-3}. [d]Error bars reflect only the error from integration, but not the error due to interference of other species, which is difficult to estimate. [e]Error bars reflect propagated errors from numbers in two rows.

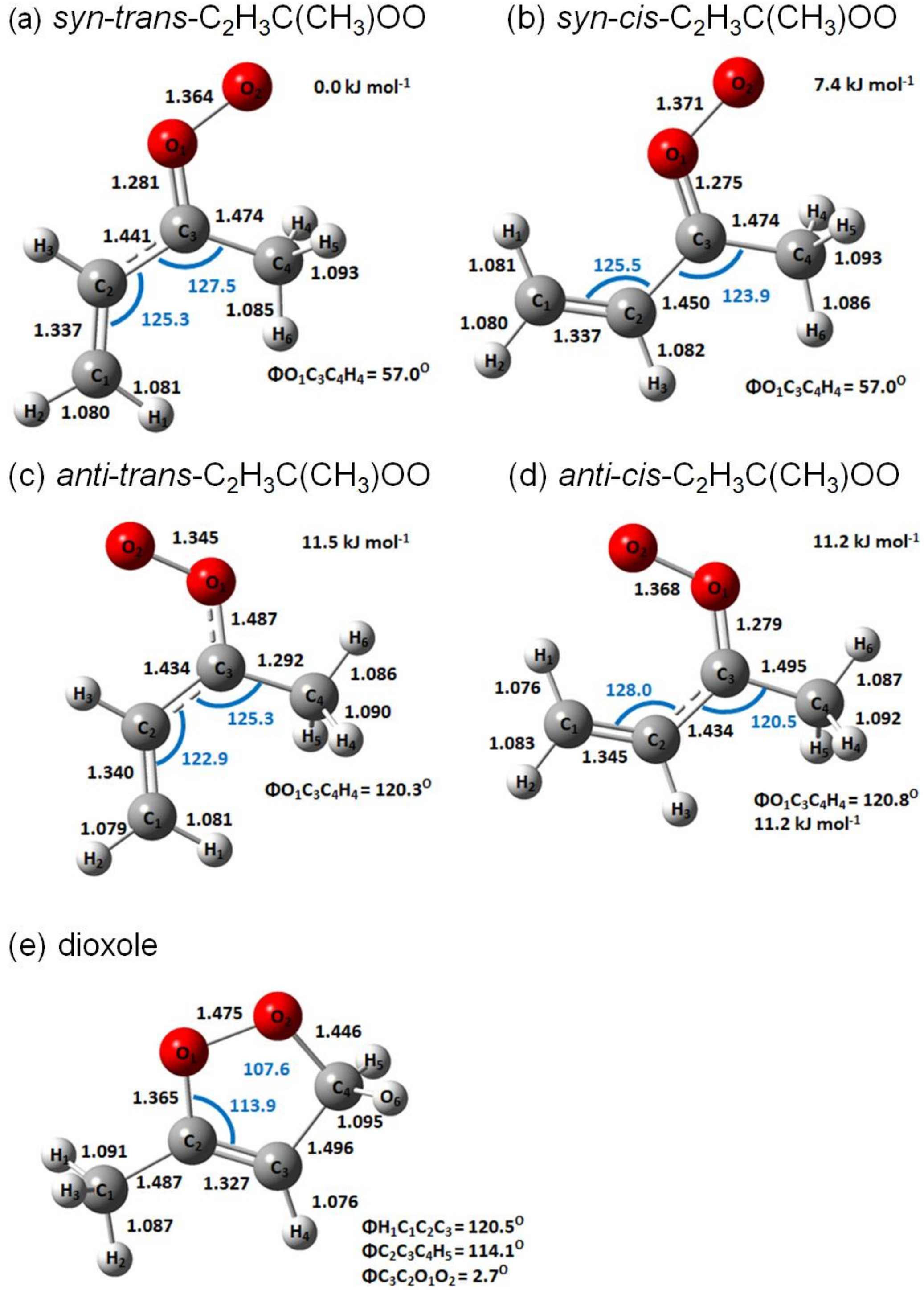

Supplementary Fig. 1 Geometries of conformers of methyl vinyl ketone oxide (MVKO) and dioxole predicted with the B3LYP/aug-cc-pVTZ method. (**a**) *syn-trans*-$C_2H_3C(CH_3)OO$ **(3)**. (**b**) *syn-cis*-$C_2H_3C(CH_3)OO$. (**c**) *anti-trans*-$C_2H_3C(CH_3)OO$. (**d**) *anti-cis*-$C_2H_3C(CH_3)OO$. (**e**) dioxole. Bond lengths (black) are in Å and angles (blue) are in degree; Φ is the dihedral angle.

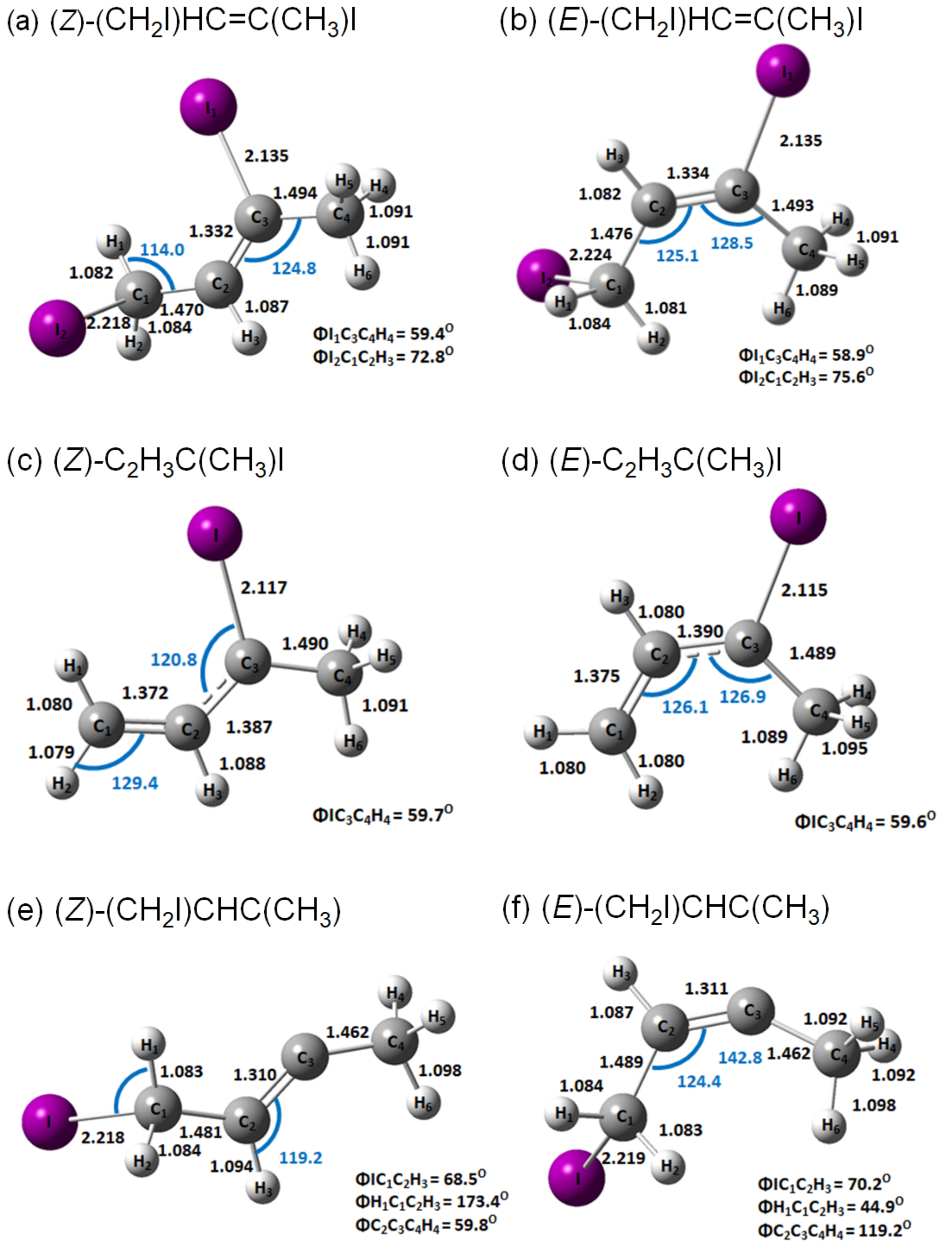

Supplementary Fig. 2 Geometries of precursors (*Z*)-/(*E*)-(CH_2I)HC=C(CH_3)I) and (*Z*)-/(*E*)- iodoalkyl radicals $C_2H_3C(CH_3)I$ and (CH_2I)CHC(CH_3) predicted with the B3LYP/aug-cc-pVTZ-pp method. (**a**) (*Z*)-(CH_2I)HC=C(CH_3)I. (**b**) (*E*)-$C_2H_3C(CH_3)I$. (**c**) (*Z*)-$C_2H_3C(CH_3)I$. (**d**) (*E*)-$C_2H_3C(CH_3)I$. (**e**) (*Z*)-(CH_2I)CHC(CH_3). (**f**) (*E*)-(CH_2I)CHC(CH_3). Bond lengths (black) are in Å and angles (blue) are in degree; Φ is the dihedral angle.

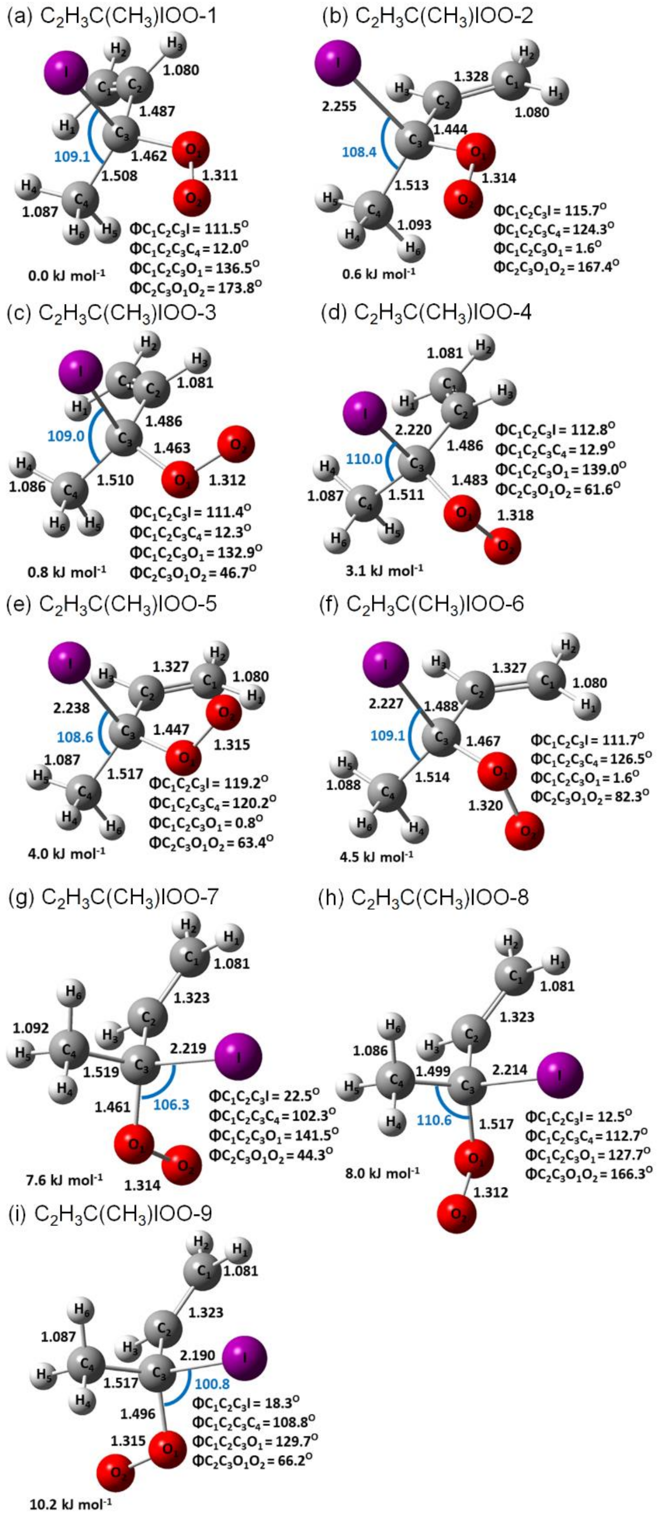

Supplementary Fig. 3 Geometries of nine conformers of iodoperoxy radicals $C_2H_3C(CH_3)IOO$ predicted with the B3LYP/aug-cc-pVTZ-pp method. Bond lengths (black) are in Å and angles (blue) are in degree; Φ is the dihedral angle.

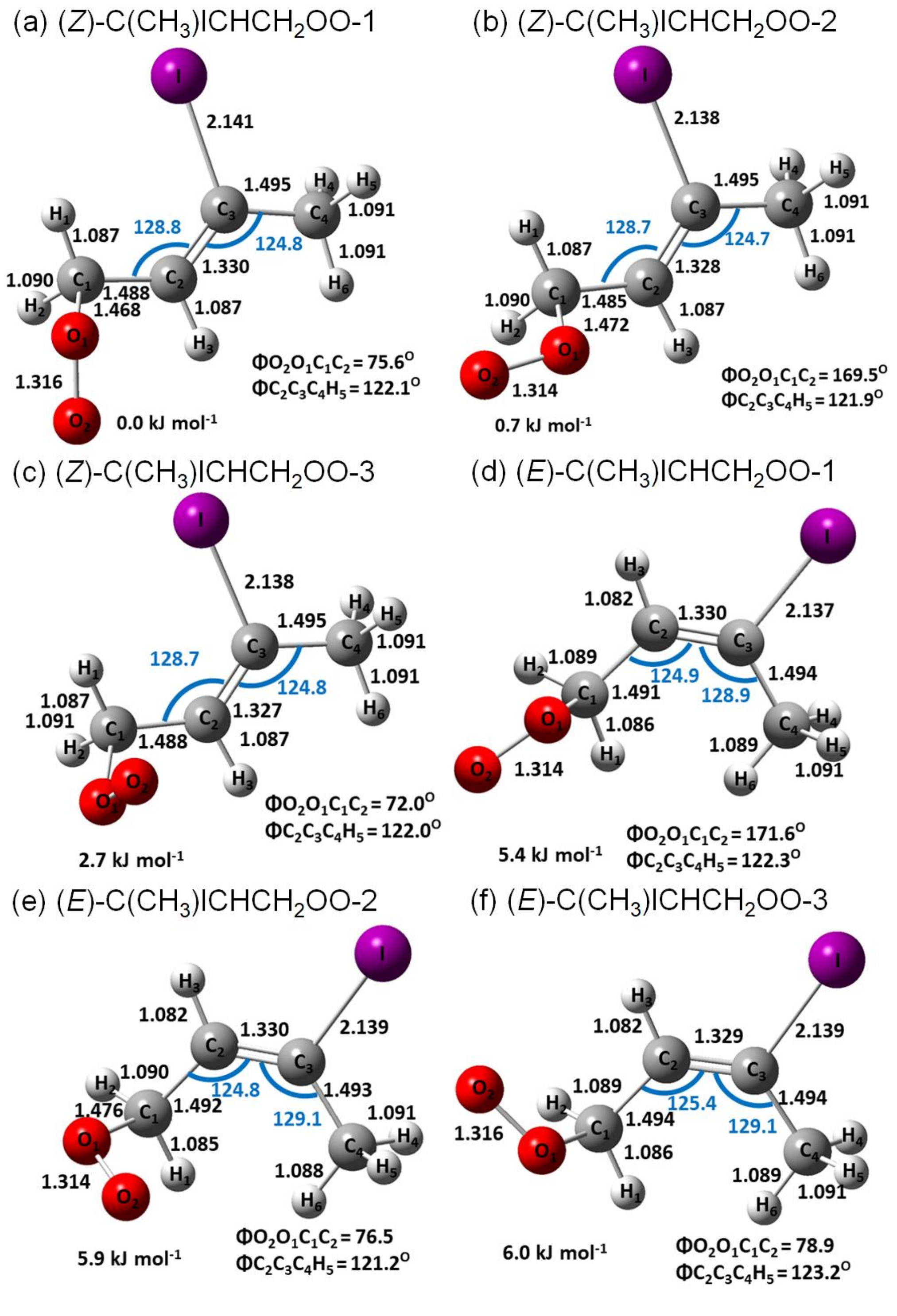

Supplementary Fig. 4 Geometries of six conformers of iodoperoxy radicals $C(CH_3)ICHCH_2OO$ predicted with the B3LYP/aug-cc-pVTZ-pp method. Bond lengths (black) are in Å and angles (blue) are in degree; Φ is the dihedral angle.

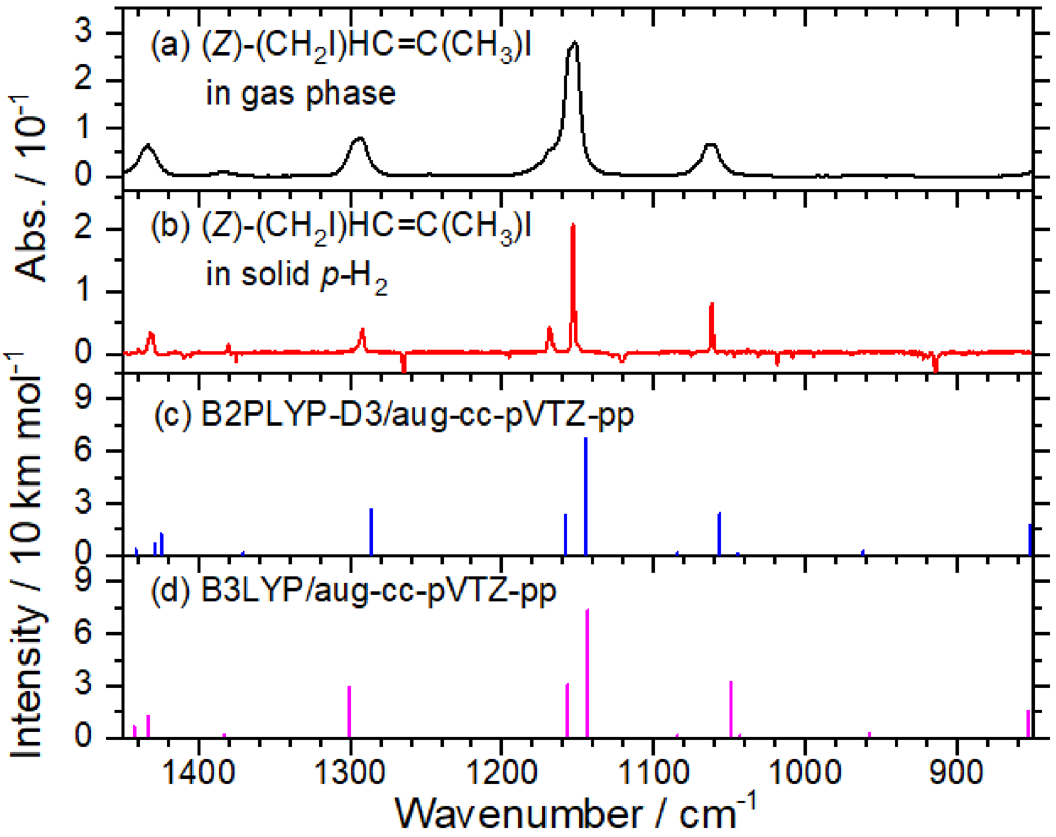

Supplementary Fig. 5 Comparison of IR spectra of (*Z*)-$CH_2IHC{=}C(CH_3)I$ (1) in the gaseous phase and in solid *p*-H_2 with quantum-chemical calculations. (**a**) Gaseous spectrum. (**b**) spectrum in solid *p*-H_2. (**c**) stick IR spectrum predicted with the B2PLYP-D3/aug-cc-pVTZ-pp method. (**d**) stick IR spectrum predicted with the B3LYP /aug-cc-pVTZ-pp method.

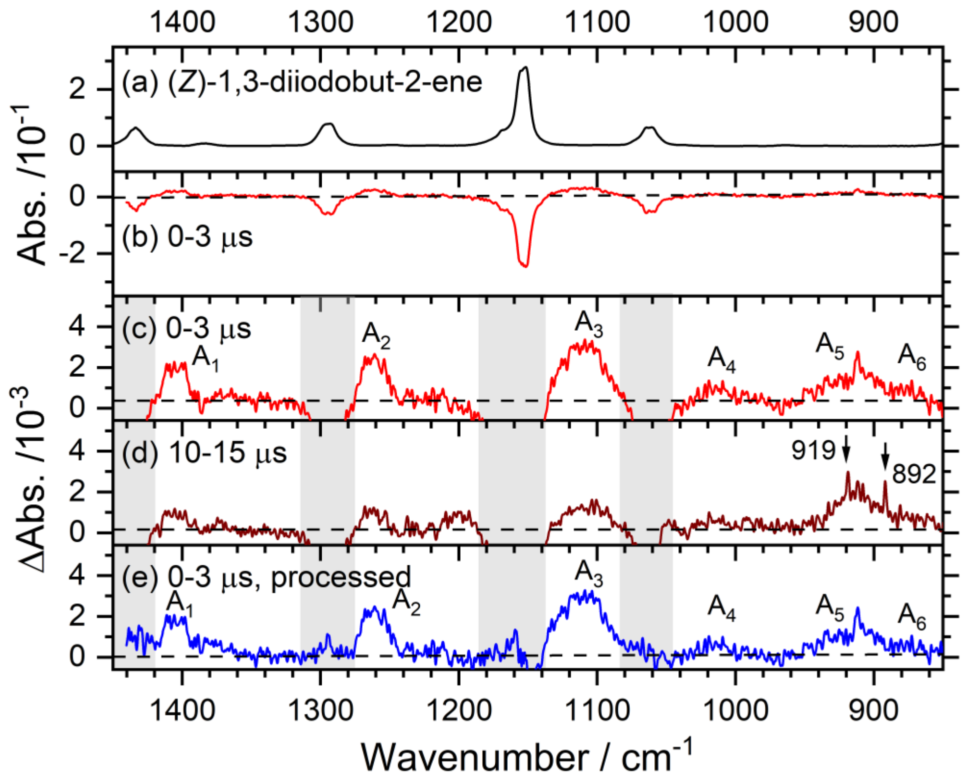

Supplementary Fig. 6 Temporal evolution of observed spectra and processed spectra in region 1450–850 cm^{-1} at resolution 1.0 cm^{-1} upon photolysis at 248 nm of a flowing mixture of (*Z*)-1,3-diiodobut-2-ene/N_2 (0.03/75 Torr) and comparison with predicted stick spectra. (**a**) Absorption spectrum before photolysis. (**b**) Difference spectrum recorded 0–3 μs after photolysis. (**c**) Expanded spectrum of (**b**) with negative bands truncated. (**d**) Expanded difference spectrum recorded 10–15 μs after photolysis. (**e**) Spectrum of (**c**) with absorption bands of precursor **(1)** added back to eliminate negative bands. Grey areas in (**c**)–(**e**) represent regions of possible interference from the absorption of precursor **(1)**. New features in group A are marked A_1–A_6.

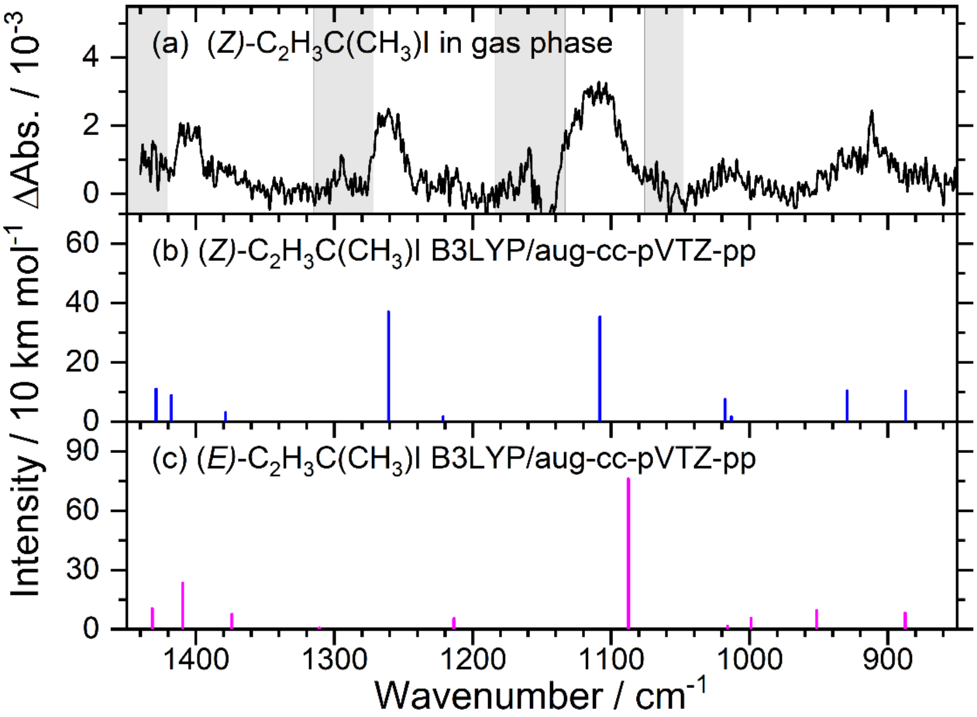

Supplementary Fig. 7 Comparison of IR spectra of (*Z*)-$C_2H_3C(CH_3)I$ (2) in the gaseous phase with quantum-chemical calculations. (**a**) (*Z*)-$C_2H_3C(CH_3)I$ in the gaseous phase. (**b**) Stick IR spectrum of (*Z*)-$C_2H_3C(CH_3)I$. (**c**) stick IR spectrum of (*E*)-$C_2H_3C(CH_3)I$ predicted with the B3LYP/aug-cc-pVTZ-pp method. Grey areas represent regions of possible interference from absorption of the parent molecules.

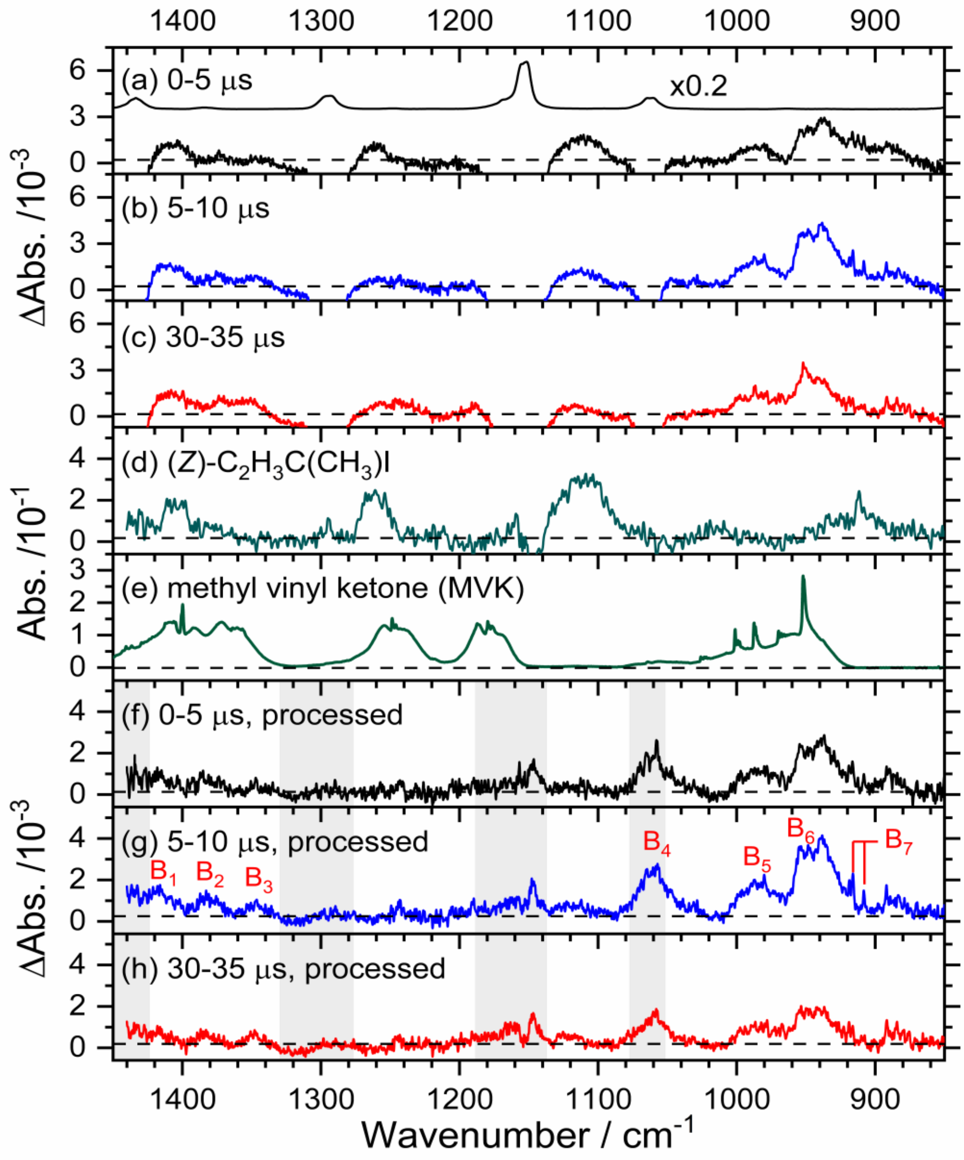

Supplementary Fig. 8 Temporal evolution of observed and processed spectra in region 1450–850 cm^{-1} at resolution 0.5 cm^{-1} upon photolysis at 248 nm of a flowing mixture of (*Z*)-1,3-diiodo-but-2-ene/O_2 (0.04/35 Torr). Difference spectra recorded 0–5 μs (**a**), 5–10 μs (**b**), and 30–35 μs (**c**) after irradiation; the absorption of the precursor is shown on a reduced scale and shifted upward in (**a**). (**d**) Spectrum of (*Z*)-$C_2H_3C(CH_3)I$ **(2)** taken from Supplementary Figure 6(e). (**e**) Absorption spectrum of methyl vinyl ketone (MVK); (**f**)–(**h**) processed spectra of (**a**)–(**c**) with bands of **(2)** and MVK removed and those of the precursor **(1)** added back. Grey areas represent regions of possible interference from absorption of the parent molecules. New features are marked B_1–B_7 in (**g**).

(a) Major resonance structures of MVKO

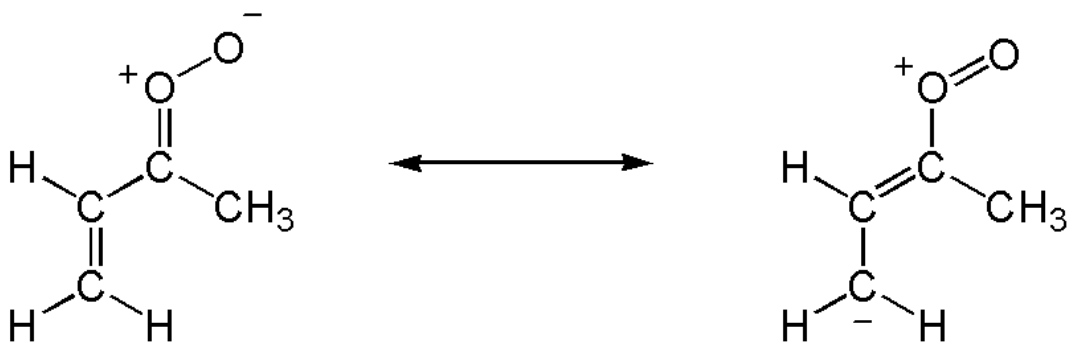

(b) Frontier-orbital diagrams showing the delocalization over the CCCOO skeleton

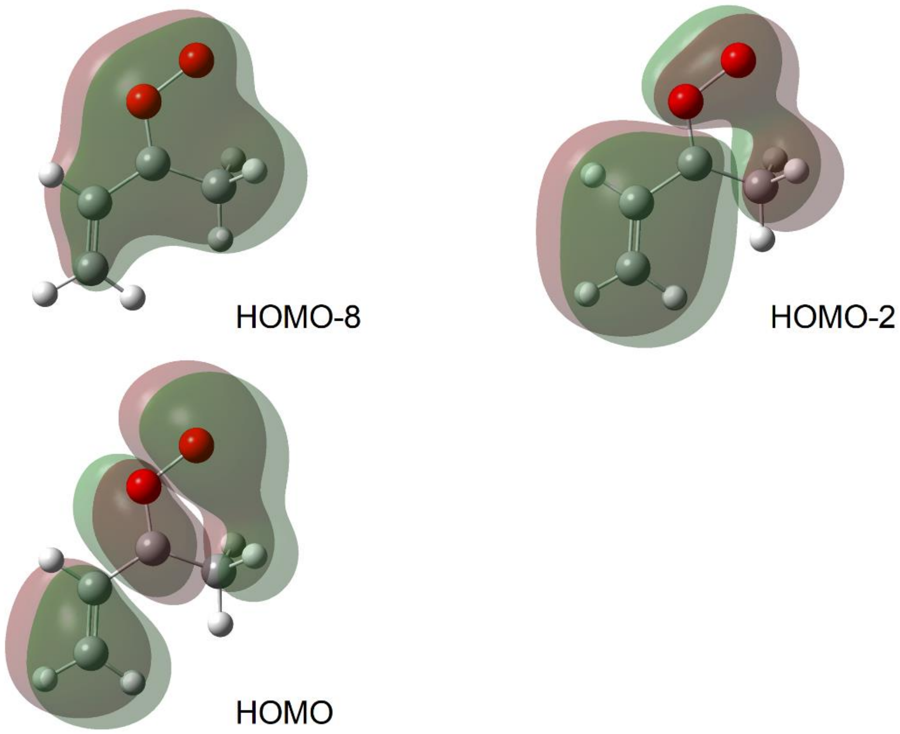

Supplementary Fig. 9 Resonance structures and frontier orbitals of *syn-trans*-MVKO. The electron density isovalues are 0.01 e/Å^3.

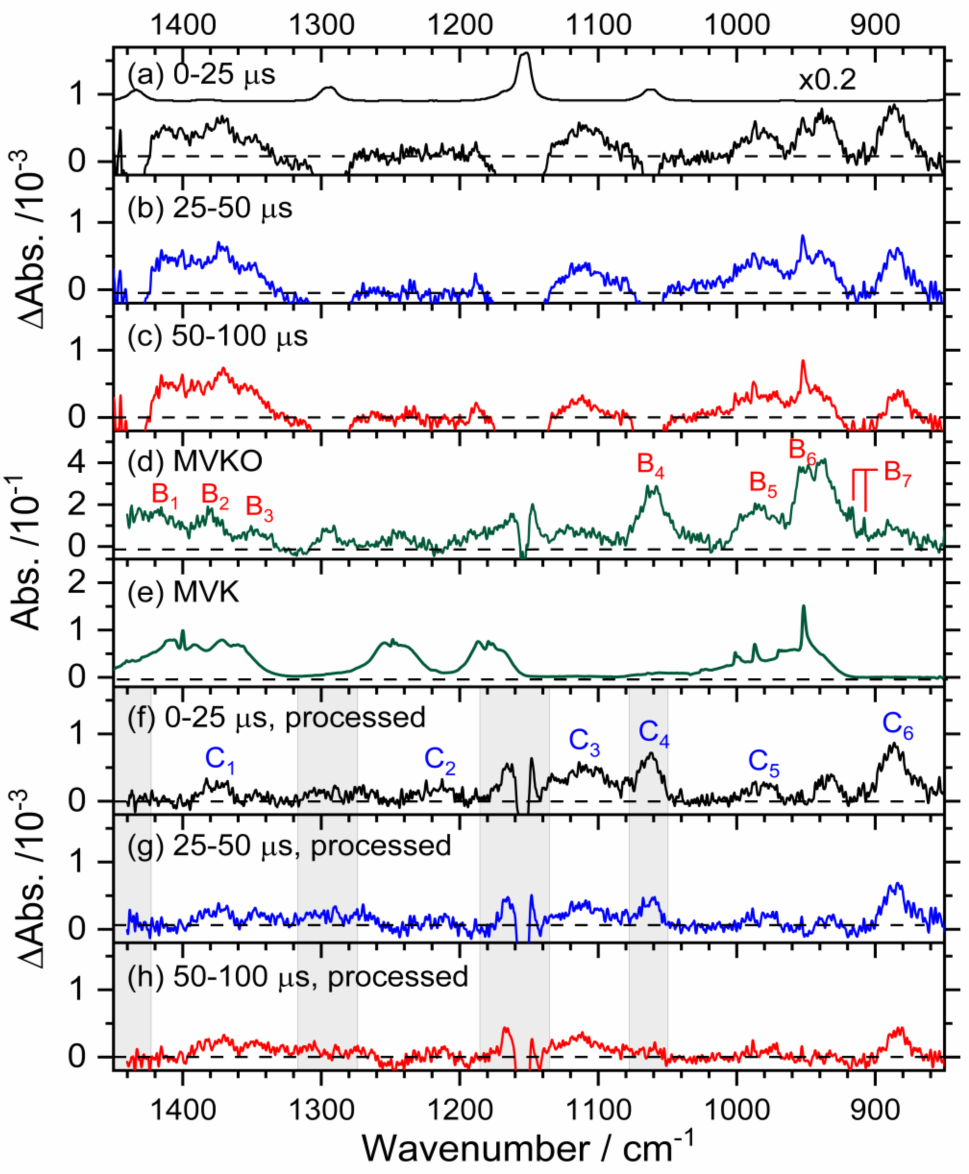

Supplementary Fig. 10 Temporal evolution of observed and processed spectra in region 1450–850 cm^{-1} at resolution 1.0 cm^{-1} upon photolysis at 248 nm of a flowing mixture of (*Z*)-1,3-diiodo-but-2-ene/O_2 (0.042/347 Torr). Difference spectra recorded 0–25 μs (**a**), 25–50 μs (**b**), and 50–100 μs (**c**) after irradiation; the absorption of the precursor is shown on a reduced scale in (**a**). (**d**) Spectrum of MVKO **(3)** taken from Figure 3(a) of main text. (**e**) Absorption spectrum of methyl vinyl ketone (MVK). (**f**)–(**h**) processed spectra of (**a**)–(**c**) with bands of **(3)** and MVK removed and those of the precursor **(1)** added back. Grey areas represent regions of possible interference from absorption of the parent molecules. New features are marked C_1–C_6 in (**f**).

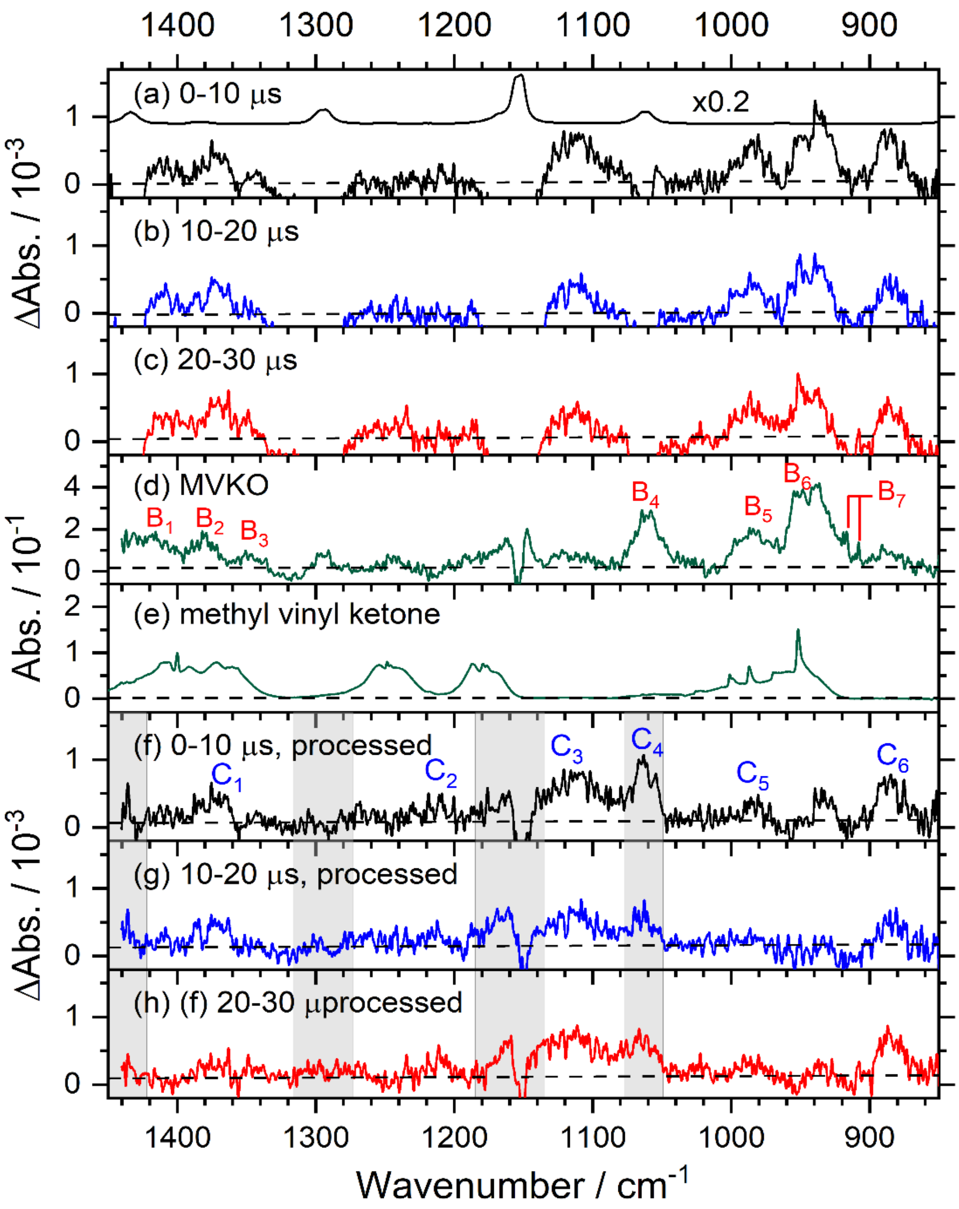

Supplementary Fig. 11 Temporal evolution of observed and processed spectra in region 1450–850 cm^{-1} at resolution 1.0 cm^{-1} upon photolysis at 248 nm of a flowing mixture of (Z)-1,3-diiodo-but-2-ene/O_2 (0.035/236 Torr). Difference spectra recorded 0–10 μs (**a**), 10–20 μs (**b**), and 20–30 μs (**c**) after irradiation; the absorption of the precursor is shown on a reduced scale in (**a**). (**d**) Spectrum of MVKO **(3)** taken from Figure 3(a) of main text. (**e**) Absorption spectrum of methyl vinyl ketone (MVK). (**f**)–(**h**) processed spectra of (**a**)–(**c**) with bands of **(3)** and MVK removed and those of the precursor **(1)** added back. Grey areas represent regions that might suffer interference from absorption of the parent molecules. New features are marked C_1–C_6 in (**f**).

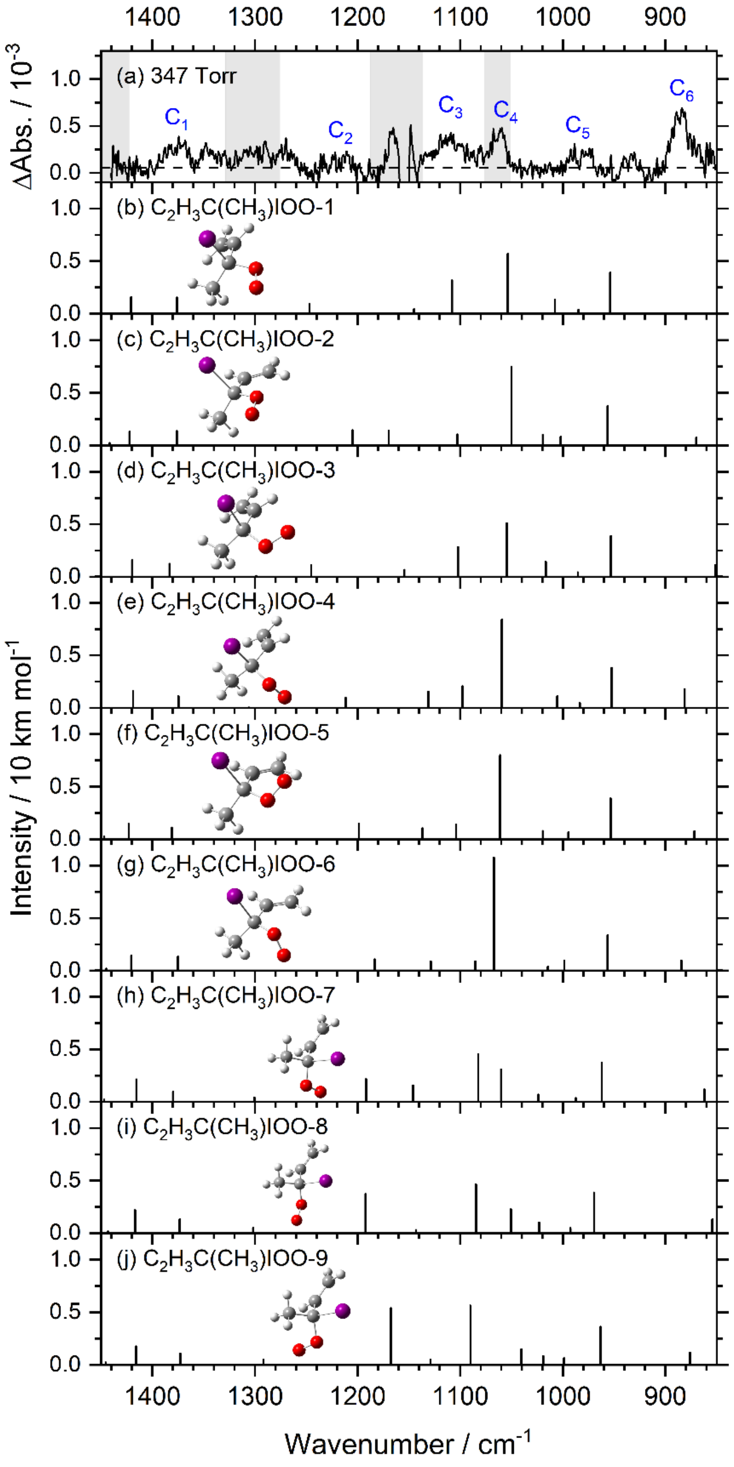

Supplementary Fig. 12 Comparison of bands in group C with stick IR spectra of nine conformers of iodoperoxy radical $C_2H_3C(CH_3)IOO$. (**a**) Spectrum taken from Supplementary Figure 10(g); grey areas represent regions of possible interference from absorption of the precursor. (**b**)–(**j**) Stick spectra of nine conformers simulated according to scaled harmonic vibrational wavenumbers and IR intensities calculated with the B3LYP/aug-cc-pVTZ-pp method.

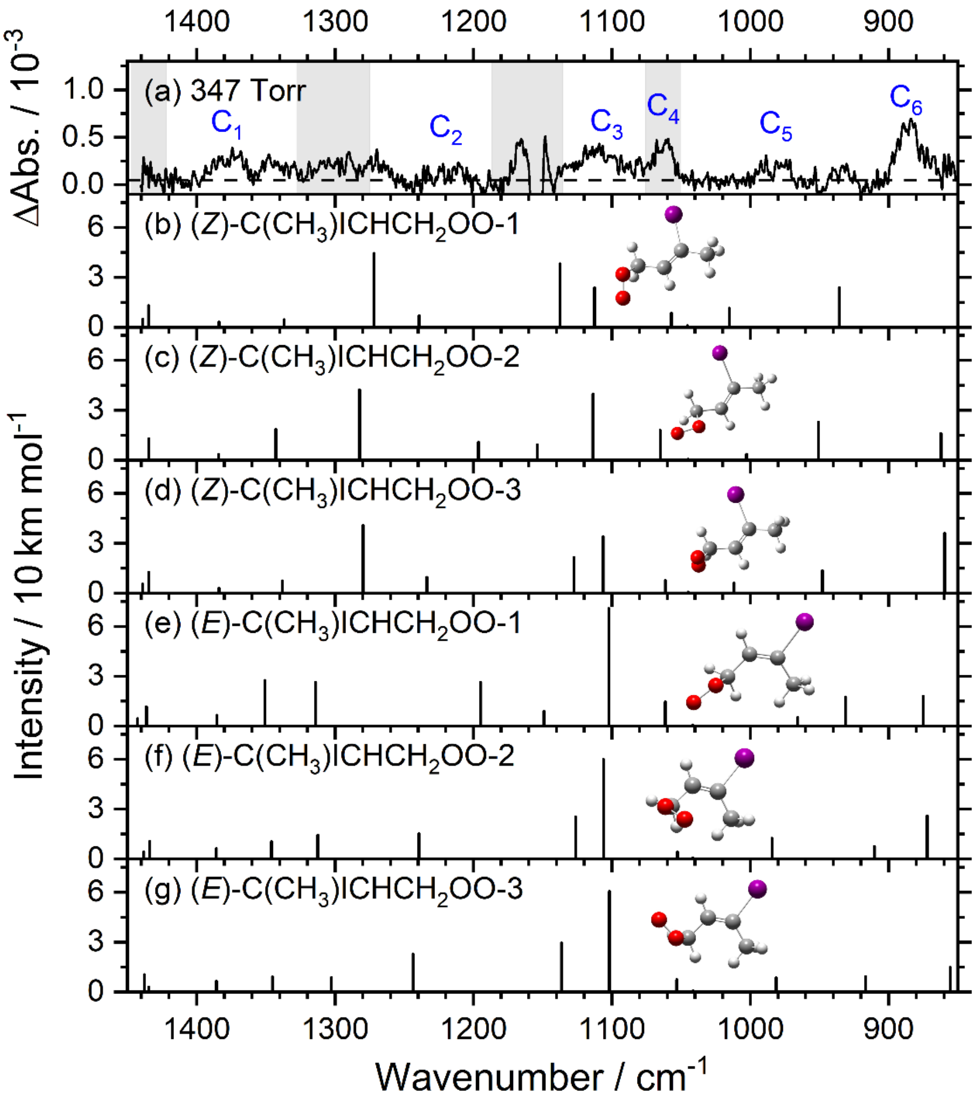

Supplementary Fig. 13 Comparison of bands in group C with stick IR spectra of six conformers of iodoperoxy radical $C(CH_3)ICHCH_2OO$. (**a**) Spectrum taken from Supplementary Figure 10(g); grey areas represent regions of possible interference from absorption of the precursor. (**b**)–(**g**) Stick spectra of six conformers simulated according to scaled harmonic vibrational wavenumbers and IR intensities calculated with the B3LYP/aug-cc-pVTZ-pp method.

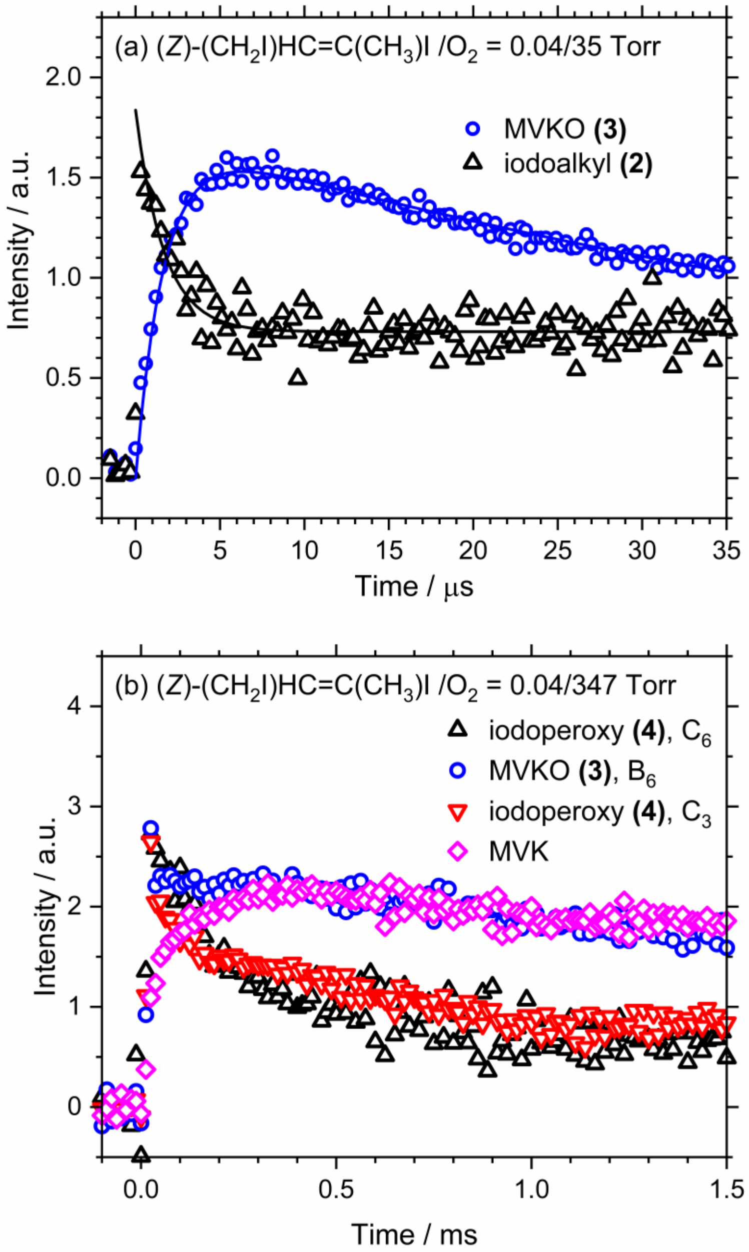

Supplementary Fig. 14 Temporal profiles of species upon photolysis at 248 nm of a mixture of (*Z*)-(CH_2I)HC=C(CH_3)I (0.04 Torr) and O_2 (35 Torr) and a mixture of (*Z*)-(CH_2I)HC=C(CH_3)I (0.04 Torr) and O_2 (347 Torr). (**a**) Criegee intermediate MVKO **(3)** integrated over region 920–960 cm^{-1} (blue circles, B_6 band) and iodoalkyl radical **(2)** integrated over region1260–1270 cm^{-1} (black triangles). (**b**) MVKO **(3)** (blue circles), iodoperoxy adduct **(4)** integrated over 1100–1120 cm^{-1} (black triangle, C_3 band) and 862–907 cm^{-1} (red inverted triangle, C_6 band), and methyl vinyl ketone (MVK) integrated over 1250–1270 cm^{-1} (pink diamonds).

Supplementary References

1. Haupa, K. A., Chen, K.-P., Li, Y.-K. & Lee, Y.-P. Infrared spectra of (*Z*)- and (*E*)-•$C_2H_3C(CH_3)I$ radicals produced upon photodissociation of (*Z*)- and (*E*)-$(CH_2I)HC{=}C(CH_3)I$ in solid *para*- hydrogen. *J. Phys. Chem. A* **124**, 5887−5895 (2020).

2. Barber, V. P., Pandit, S., Green, A. M., Trongsiriwat, N., Walsh, P. J., Klippenstein, S. J. & Lester, M. I. Four-carbon Criegee intermediate from isoprene ozonolysis: methyl vinyl ketone oxide synthesis, infrared spectrum, and OH production. *J. Am. Chem. Soc.* **140**, 10866−10880 (2018).

3. Western, C. M. *PGOPHER, a Program for Simulating Rotational Structure* (University of Bristol UK, 2010) version 10.1.183 http://pgopher.chm.bris.ac.uk.

4. Huang, Y.-H., Chen, L.-W. & Lee, Y.-P. Simultaneous infrared detection of the ICH_2OO radical and Criegee intermediate CH_2OO: The pressure dependence of the yield of CH_2OO in the reaction $CH_2I + O_2$. *J. Phys. Chem. Lett.* **6**, 4610−4615 (2015).

5. Lin, Y.-H., Li, Y.-L., Chao, W., Takahashi, K. & Lin, J. J.-M. The Role of the iodine-atom adduct in the synthesis and kinetics of methyl vinyl ketone oxide— a resonance-stabilized Criegee intermediate, *Phys. Chem. Chem. Phys.* **22**, 13603−13612 (2020).

6. Chung, C.-A., Su, J.-W. & Lee, Y.-P. Detailed mechanism and kinetics of the reaction of Criegee intermediate CH_2OO with HCOOH investigated via infrared identification of conformers of hydroperoxymethyl formate and formic acid anhydride. *Phys. Chem. Chem. Phys.* **21**, 21445−21455 (2019).
